# Transcription instability in high‐risk neuroblastoma is associated with a global perturbation of chromatin domains

**DOI:** 10.1002/1878-0261.12139

**Published:** 2017-10-10

**Authors:** Carlo Zanon, Gian Paolo Tonini

**Affiliations:** ^1^ Neuroblastoma Laboratory Pediatric Research Institute, Citta' della Speranza Padua Italy

**Keywords:** chromatin structural domain, superenhancer, transcriptional instability

## Abstract

Chromosome instability has a pivotal role among the hallmarks of cancer, but its transcriptional counterpart is rarely considered a relevant factor in cell destabilization. To examine transcription instability (TIN), we first devised a metric we named TIN index and used it to evaluate TIN on a dataset containing more than 500 neuroblastoma samples. We found that metastatic tumors from high‐risk (HR) patients are characterized by significantly different TIN index values compared to low/intermediate‐risk patients. Our results indicate that the TIN index is a good predictor of neuroblastoma patient's outcome, and a related TIN index gene signature (TIN‐signature) is also able to predict the neuroblastoma patient's outcome with high confidence. Interestingly, we find that TIN‐signature genes have a strong positional association with superenhancers in neuroblastoma tumors. Finally, we show that TIN is linked to chromatin structural domains and interferes with their integrity in HR neuroblastoma patients. This novel approach to gene expression analysis broadens the perspective of genome instability investigations to include functional aspects.

**Abbreviations**
AUC: area under the curve
CED: coordinated expression domains
HR: high‐risk
LIR: low/intermediate‐risk
SE: superenhancers
TAD: topologically associating domains
TIN: transcription instability

## 1 Introduction

Chromosome instability is a hallmark of cancer (Hanahan and Weinberg, 2001), but its transcriptional counterpart is seldom taken into consideration as a relevant factor in cell destabilization. In the present work, we systematically approach the study of transcription instability (TIN) in neuroblastomas as an integral part of genome instability. Neuroblastomas can occur as a localized or metastatic tumor (Maris *et al*., 2007). Metastatic neuroblastoma predominately occurs in patients older than 1 year of age as a very aggressive stage 4 disease. Stage 4S metastatic neuroblastoma, however, occurs in infants and results in a good outcome for approximately 70% of cases. Localized neuroblastomas are less aggressive and include stages 1, 2, and 3 that have a more favorable outcome of 65–98%. Localized neuroblastoma tumors are characterized by several numerical but few structural chromosomal variations (Coco *et al*., 2012; Scaruffi *et al*., 2007; Schleiermacher *et al*., 2010). In contrast, stage 4 tumors have more structural than numerical alterations. Stage 4S tumors have intermediate structural and numerical alterations. These findings imply that neuroblastoma cells have a significant amount of genome instability. How this chromosome disruption is reflected in gene transcription is still unclear. Indeed, gene expression studies show that several genes are abnormally expressed in neuroblastoma cells (Tonini and Romani, 2003). Gene expression profiles of neuroblastoma cells have been investigated in numerous studies and have resulted in a number of gene expression signatures being used to evaluate the patient's risk (De Preter *et al*., 2010; Oberthuer *et al*., 2010; Vermeulen *et al*., 2009). This further indicates that an ensemble of genes, rather than one single gene, are abnormally expressed in neuroblastoma cells, and this contributes to tumor development.

Today, thanks to high‐throughput transcriptome technologies, large amounts of data on neuroblastoma cells are publicly available. In the present work, we introduce the concept of global TIN as a result of unusual transcriptome activity as quantified by the TIN index metric. We observed an overall increase in TIN in poorer outcome samples in association with the presence of superenhancers (SE) and correlated with global stochastic changes in the whole transcriptome. Our observations fit well with recently reported results in neuroblastoma cells by Valentijn *et al*. (2015) and Peifer *et al*. (2015), where both authors showed, by whole‐genome sequencing, that regions in the vicinity of the TERT gene are prone to rearrangements in high‐risk (HR) neuroblastomas. These genomic alterations were instrumental in positioning SE close to the breakpoints and were eventually associated with TERT overexpression. Moreover, most of the transcripts contributing to the TIN are preferentially located within coordinated expression domains (CEDs), where neighboring genes show coordinated expression (Lercher *et al*., 2002; Woo *et al*., 2010), and these, in their turn, show a genome‐wide tendency to colocalize with regulatory features like enhancers (Acemel *et al*., 2016; Chepelev *et al*., 2012; de Laat and Duboule, 2013; Tang *et al*., 2015).

In conclusion, our study introduces, for the first time, the concept of TIN in neuroblastoma cells and shows that the global transcription alteration in neuroblastoma cells is physically associated with CED and SE.

## 2 Material and methods

### 2.1 Transcription Instability

#### 2.1.1 Gene expression data

Gene expression values of 504 NB samples from the E‐MTAB‐161 dataset (ArrayExpress database at EMBL) were used in this study. Primary data were retrieved from the database entry as preprocessed normalized data. The corresponding probes' sequences were remapped onto the hg19 reference genome using bowtie2 software (Langmead and Salzberg, 2012) and only high‐confidence, unambiguous mapping probes were retained for further analyses. The preprocessed normalized expression intensities of the probes were then collapsed onto the corresponding hg19 RefSeq gene, using the median signal intensity in case multiple probes mapped to the same gene. Gene entries with more than 25% of their values missing were discarded. Gene expression values were rank‐ordered using the function ‘rank’ from the package ‘base’ of the r statistical software (R core team, 2013). The gene rank‐ordered entries in the data matrix were finally ordered by chromosome and their physical position according to the hg19 genome coordinates. The resulting gene‐centered data matrix was then used for all subsequent analyses.

#### 2.1.2 Clinical data

**Figure 1:**
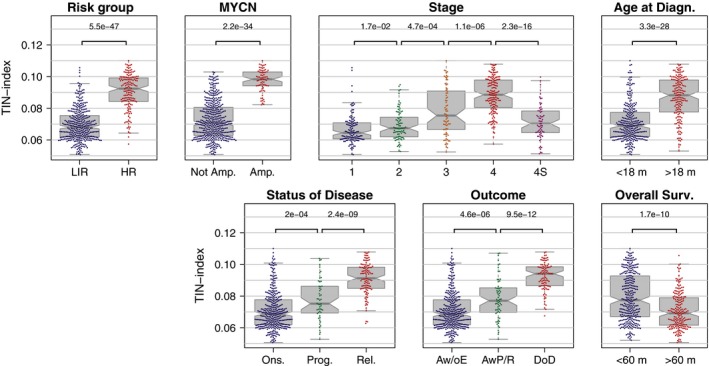
TIN index distribution. Boxplots representing the distribution of TIN index values (calculated using stage 1 samples as a reference) stratified by clinical feature. Wilcox test *P*‐values of statistically significant differences are reported on top. Not Amp., not amplified; Amp., amplified; Ons., onset; Prog., progression; Rel., relapse; Aw/oE, alive without event; AwP/R, alive with progression or relapse; DoD, dead of disease; m, months.

Available clinical information about the sample in the database was used to define two risk groups according to the High Risk Neuroblastoma Study 1.7 of SIOP‐Europe (SIOPEN) specifications. A HR group consisted of samples at stages 2, 3, 4, and 4S with *MYCN* gene amplification or stage 4 samples with an age at diagnosis over 12 months of age. As a consequence, the remaining samples were grouped into the ‘low/intermediate‐risk’ (LIR) group. All the functions and packages mentioned hereafter and used for analysis and graphical representation are tools of the r statistical software, unless otherwise specified. Stratification of relevant clinical features was represented using the functions ‘boxplot’ and ‘beeswarm’ from the packages ‘graphics’ and ‘beeswarm’, respectively; the *P*‐values reported in Fig. 1 and Fig. S1 were calculated using the function ‘wilcox.test’ from the package ‘stats’.

### 2.2 TIN index and TIN‐signature

For each sample, we defined the TIN index of a gene (gene‐wise TIN index) as the squared deviation of the gene expression value from its expression value in a reference sample. The reference sample could be a single specimen or a set of samples whose expression values will be reduced to a single estimate through their average or their median; in cases of no clear consensus about what a good reference for the study should be, the entire set of samples could also be considered. Indeed in the present study, lacking a common consent on what a good reference would be for a neuroblastoma specimen, we first used all the samples as reference, as the dataset composition in terms of tumors clinical characteristics mirrored the observed prevalence in the population**.** Although there is a slight bias of the stage distribution toward favorable cases, these distributions appear to be substantially in line with their general prevalence in the population according to Haupt *et al*. (2010) (Table S1). The global TIN index, which we simply refer to as the TIN index throughout this work, is the average of all the gene‐wise TIN indexes (for all the genes considered) in a given sample:

(1) $$\text{TIN}\;\text{index}=\frac{\sum_{i=1}^{N}{\left({\text{expr}}_{i}-{\text{Expr}}_{i}\right)}^{2}}{N}$$

In the formula, for each gene *i*, Expr_*i*_ represents the average expression value of that gene in the reference samples; expr_*i*_ is its expression in a single sample; and *N* is the total number of genes considered in the analysis. The TIN index is therefore a metric related to each sample.

We then calculated the squared Pearson correlations between the expression of each probe and the TIN index across all the samples in the dataset with the aim of evaluating both positive and negative correlations. The resulting correlation values were then ranked and the probes unambiguously mapped to known RefSeq genes with squared Pearson correlations above 0.425 were then included in a shortlist named ‘TIN‐signature’. The correlation threshold was identified by selecting the top 2.5% of the squared Pearson correlation values which allowed the selection of some hundred genes (namely 184), a number that granted an informative pathway analysis aimed at pinning down important aspects underlying the TIN index. Nine probes in the dataset (namely A_32_P440054, A_32_P526498, A_32_P6008, A_32_P73532, A_32_P73535, Hs135492.1, Hs22245.1, Hs23691.1, and RNU6‐71P) were not unambiguously mapped to known RefSeq genes therefore excluded from the TIN‐signature. An unsupervised hierarchical clustering using the TIN‐signature genes was performed using the function ‘heatmap.plus’ from the package ‘heatmap.plus’ and the ‘minkowski’ distance and ‘ward.D2’ clustering methods. The resulting hierarchical heatmaps were then generated using the function ‘heatmap3’ from the package ‘heatmap3’.

Receiver operating characteristic (ROC) and Kaplan–Meier curves were calculated on the validation subset and were generated using the functions ‘roc’ and ‘survfit’ from the packages ‘pROC’ and ‘survival’, respectively.

We tested the significance of the difference between each pair of ROC curve AUCs present in Fig. 3A by means of the ‘roc.test’ function within the ‘pROC’ r package (using either the default ‘delong’ or the ‘bootstrap’ methods, both with 10 000 bootstrap replicates).

The multivariate analysis was performed applying the Cox proportional hazard model using the ‘coxph’ function within the ‘survival’ package of r statistical software.

Pathway enrichment analysis was carried out using egan software (v1.5); functional interaction networks were generated using egan (version 1.4; Paquette and Tokuyasu, 2010). Overrepresented association nodes were tested by both Fisher's exact test and Westfall–Young (Westfall *et al*., 1993) minP with 10 000 permutations.

### 2.3 Chromatin domains and transcription

**Figure 2:**
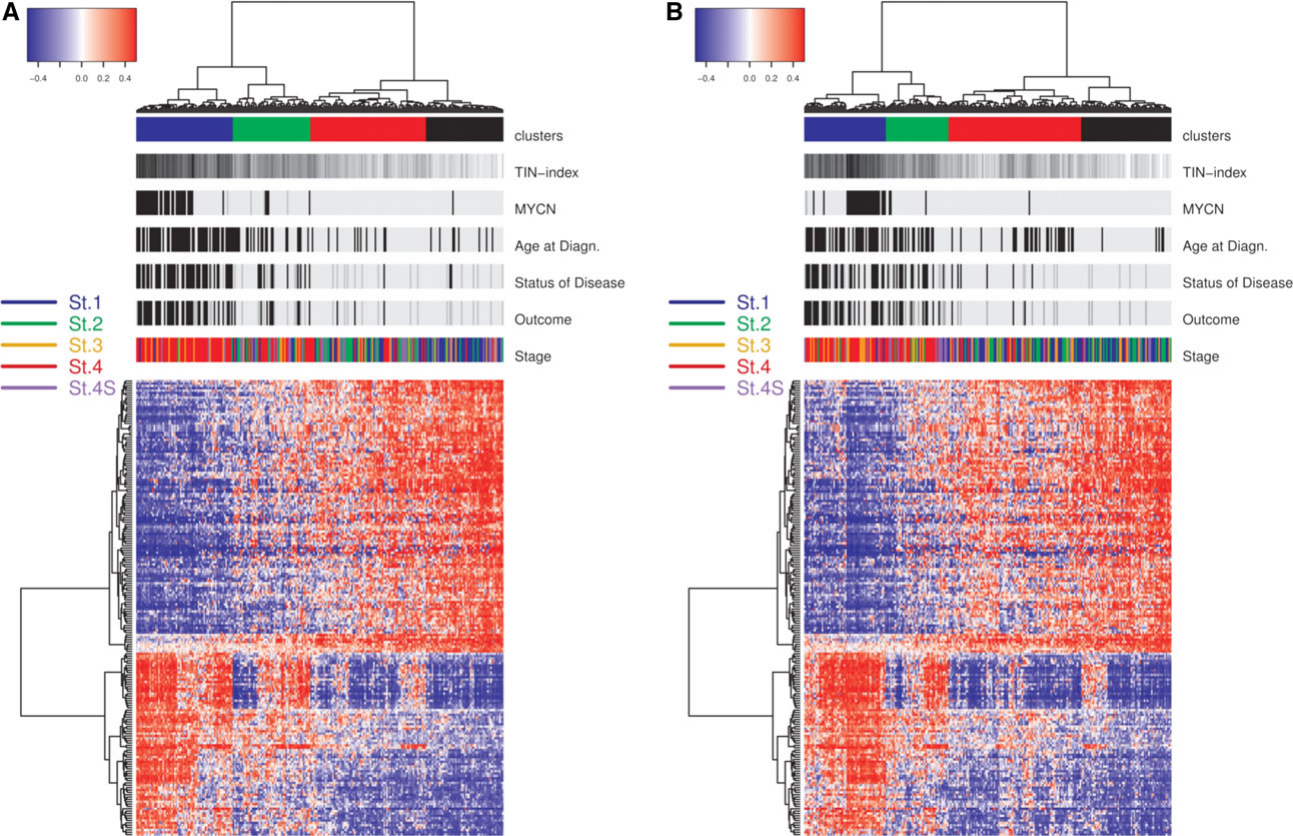
Unsupervised clustering of the training and validation sets. Heatmaps of the unsupervised clustering in the training (A) and validation (B) sets. We selected 184 genes with the highest correlation with the TIN index values across the training set for an unsupervised clustering and then tested them in the validation set. Relevant clinical features, TIN index, and the identified clusters are reported on top; black bars represent unfavorable scores, while light gray bars represent favorable scores. Black bars for the ‘Age at Diagn.’ identify cases with ages over 60 months at diagnosis. Color code for the staging is shown in the legend on the left of each panel.

To graphically represent the correlation patterns of genome‐ordered expression values, the corresponding correlation matrixes were first 2D‐smoothed using the ‘interp.loess’ function from the package ‘tgp’. This process allowed us to capture and visualize the correlations among average gene expression levels of entire regions of the genome as ‘plaid patterns’ (Fig. 2, Figs S2 and S3). The upper triangular part of the resulting 2D‐smoothed correlation matrices was then extracted using the function ‘upper.tri’ from the ‘base’ package, rotated 45° counterclockwise using the ‘grid.raster’ function from the ‘raster’ package and plotted as heatmaps using the ‘heatmap3’ function from the ‘heatmap3’ package.

### 2.4 Colocalizing genomic features

Colocalization analyses were carried out to ascertain statistical enrichment in overlap between TIN‐signature genes and CEDs, as well as between these two series and functionally meaningful genome features in neuroblastoma cells such as SE, CTCF binding regions, and early/late‐replicating intervals. We did this by comparing the counts of the overlap of occurrences between TIN‐signature genes and CEDs with the expected overlap count generated after randomly shuffling the positions of the second series and testing the results using the two‐tailed test of proportions (using the ‘pnorm’ function from ‘stats’ package). The neuroblastoma features derived from the above analysis are the positions of TIN‐signature genes, the CED's cores, and the edge regions, whereas the genomic features of neuroblastoma cell lines are the CTCF and cohesin complex binding regions and the early and late replication timing regions of the SK‐N‐SH cell line along with the clusters of the H3K27ac peaks as defined according to Fig. 1 of Pott and Lieb (2014) and to Hnisz *et al*. (2013) (marker of SE, open chromatin) of the Kelly, SH‐SY5Y, NB1, NB2 and NB3 cell lines and the H3K4me3 peaks (marker of compacted, silenced chromatin) of BE(2)‐C cell line. Bed files with the positions and spans of the top ranking TIN‐signature genes and CEDs core and edge regions were produced using hg19 as the reference genome. CED position and spans were defined using the directionality index (Dixon *et al*., 2012), with cores identified by the central quartiles of the CED interval and the edge the terminal interval. The bed files with the features positions were generated from bigWig track files.

## 3 Data accessibility

The preprocessed normalized expression data of 504 neuroblastoma samples belong to the E‐MTAB‐161 dataset, retrieved from the following source: https://www.ebi.ac.uk/arrayexpress/files/E-MTAB-161/E-MTAB-161.processed.1.zip.

The data used for the colocalization studies were retrieved from the following sources: neuroblastoma cell lines CTCF sites from the GEO sample accession GSM1003633, UCSC accession wgEncodeEH003371; cohesin complex from the GEO sample accession GSM1003627, UCSC accession wgEncodeEH003377; the early and late replication timing regions form GEO sample accession GSM923441, UCSC accession wgEncodeEH002384 for the SK‐N‐SH cell; the clusters of the H3K27ac peaks of the Kelly cell line from GEO sample accession GSM1532401, of SHSY5Y cell line from GEO sample accession GSM1532408, of NB1 cell line from GEO sample accession GSM1532414, of NB2 cell line from GEO sample accession GSM1532415 and of NB3 cell line from GEO sample accession GSM1532417; the H3K4me3 peaks of BE(2)‐C cell line from GEO sample accession GSM945241, UCSC accession wgEncodeEH001906.

## 4 Results

### 4.1 Transcription instability

In this study, we analyzed a publicly available dataset of gene expression profiles of 504 neuroblastoma tumor samples (E‐MTAB‐161 dataset, see Material and methods, subsection 2.1.1) annotated with the most relevant clinical information (Fig. S4). To quantify the TIN in these neuroblastoma samples, we defined a metric named ‘TIN index’, as a measure of the global transcriptional alteration (Material and methods, subsection 2.2 for details). As the dataset was lacking samples to be used as reference, we started by measuring, in each sample, the deviation of each gene expression from its average value in the entire dataset, provided that the dataset composition in terms of clinical features reflected the observed prevalence in the population (Table S1). We found that the TIN is less perturbed in LIR tumors, mirroring the scarcity of structural aberrations that are associated with unfavorable outcomes in the more aggressive HR neuroblastoma patients (Janoueix‐Lerosey *et al*., 2009).

We stratified the TIN index by main clinical and biological features and observed that it strongly correlates with the patient's clinical stage, age at diagnosis, 5‐year overall survival, and *MYCN* (single gene copy versus amplified) status of the tumor (Fig. S1). High TIN index values are strongly associated with stage 4 tumors, the occurrence of *MYCN* gene amplification, and the patient's age (> 18 months) at the time of diagnosis. This finding indicates that stage 4 tumors have a significant amount of transcriptional deregulation compared to localized tumors and suggests a correlation of TIN with high tumor aggressiveness. Of note, stage 4S tumors, although metastatic, are less aggressive than stage 4 tumors and TIN index values closer to stage 1 and 2 tumors, as expected. The TIN indexes of stage 3 tumors display more variable values compared to that of other stages, suggesting more heterogeneous transcriptional alterations (Fig. S1). Lastly, HR patients (those with tumors at stages 2, 3, 4, and 4S with *MYCN* amplification or stage 4 patients over 12 months of age) are characterized by a high TIN index compared to those found in the LIR group, confirming that elevated TIN is associated with very aggressive tumors.

To confirm and further investigate the correlation between the TIN index and patient's outcome, we adopted an additional approach for calculating the TIN index. As we are primarily focused on evaluating TIN and its role in HR neuroblastoma patients, we used the mean gene expressions of stage 1 *MYCN* nonamplified localized tumors as reference values. We also excluded a stage 2 tumor sample from further analysis as it had a TIN index value markedly out of range compared to all other samples and was thus considered to be an outlier (Fig. S1). The results of this approach perfectly reflect and strengthen our previous observations regarding the correlation between the TIN index and relevant clinical features (Fig. 1) and allow us to use this reference throughout our studies. Additionally, these results allowed us to conclude that the transcriptome in localized tumors is less perturbed than in the disseminated tumors of HR patients.

**Figure 3:**
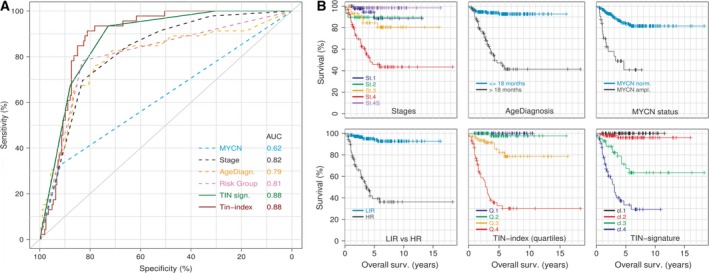
ROC and survival curves. (A) ROC curves of the following prognostic factors: *MYCN* amplification status, tumor stage, age at diagnosis, risk group, TIN‐signature, and TIN index. The corresponding AUC values for each prognostic factor are reported for comparison in the legend. (B) Panels showing the Kaplan–Meier plot of tumor stage, age at diagnosis, *MYCN* amplification status, and risk group (top row and bottom left panels) in comparison with the TIN index and TIN‐signature classifiers (bottom central and right panels). The TIN‐signature panel (bottom right) maintains the color code of the clusters identified by the unsupervised clustering as shown in the heatmaps (Fig. 2A,B).

To test the prognostic value of the TIN index, we randomly divided the dataset samples into two parts: a subset of 251 samples to be used as the training set and a second set with 252 samples to be used as the validation set. We evaluated the TIN index performance as a classification tool and compared it to other known clinical‐based classifiers using ROC curves (Fig. 3A). The TIN index shows an area under the curve (AUC) of 0.88 compared to 0.81 for the risk group, 0.82 for the clinical stage, 0.79 for the age at diagnosis, and 0.62 for the *MYCN* status, showing the potential of the TIN index as a prognostic marker. The Kaplan–Meier analysis further supported the prognostic value of all the tested factors (Fig. 3B). The previous results were further investigated through a multivariate analysis testing the above‐mentioned covariates for their independent contribution as prognostic factors, with the TIN index ranking within the top predictors, thus confirming its prognostic value (Table S2). Overall, these results show that high TIN index values are associated with unfavorable outcomes of very aggressive neuroblastomas.

To rank genes by their individual contribution to the TIN index, we next calculated the absolute Pearson correlation between the TIN index and the expression value of each gene in the previously described training set. The top ranking genes identified a TIN‐signature that can discriminate between samples from both groups into clinically meaningful clusters (Fig. 2A,B and Table S3). The corresponding TIN‐signature ROC curve shows that its prognostic value is comparable to the TIN index (Fig. 3A and Table S4). The TIN‐signature has not inferior potential for classifying patients with respect to known clinically based classifiers such as clinical stage, *MYCN* gene amplification, or age at diagnosis (Oberthuer *et al*., 2015). The corresponding survival curves confirm that the TIN‐signature is capable of discriminating between good‐ and poor‐outcome patients (Fig. 3B). Pathway analyses on the TIN‐signature genes revealed that the corresponding statistically significant enrichments show an almost exclusive connection to DNA replication‐related activities such as nucleotides metabolism, replication initiation and progression, replication stress management along with cell cycle progression and chromosome maintenance pathways (Tables S5–S8). Furthermore, the pathway enrichment analysis performed on the samples stratified by cluster groups, as defined by the unsupervised clustering using the TIN‐signature genes, revealed that the ‘better outcome’ clusters (namely the ones labeled in red and black at the top in Fig. 2) show a prevalent expression of genes related to DNA damage response, whereas the ‘worse outcome’ ones (labeled in blue and green) show a clear enrichment for many cancer‐related pathways (Table S9). These results demonstrate that the TIN index can be linked to a correlated gene expression signature with a prognostic value at least comparable to the already‐known neuroblastoma gene signatures (De Preter *et al*., 2010; Oberthuer *et al*., 2010; Vermeulen *et al*., 2009) and strongly associated with DNA replication processes.

### 4.2 Chromatin domains and transcription

It is generally assumed that coexpressed/repressed genes do not map randomly in the genome but tend to cluster in specific regions within chromosomes (Schoenfelder *et al*., 2010). A degree of correlated expression is also evident among genes across chromosomes, possibly due to their functional relationships, activity in the same pathway or protein complex, or because of their physical proximity within the interphase nucleus, as was recently shown by genome conformation capture (Hi‐C) data (Fanucchi *et al*., 2013). These observations lead to the notion that active coregulated genes and their regulatory factors cooperate preferentially via intra‐ and interchromosomal conformation interactions (Lieberman‐Aiden *et al*., 2009; Nora *et al*., 2013).

**Figure 4:**
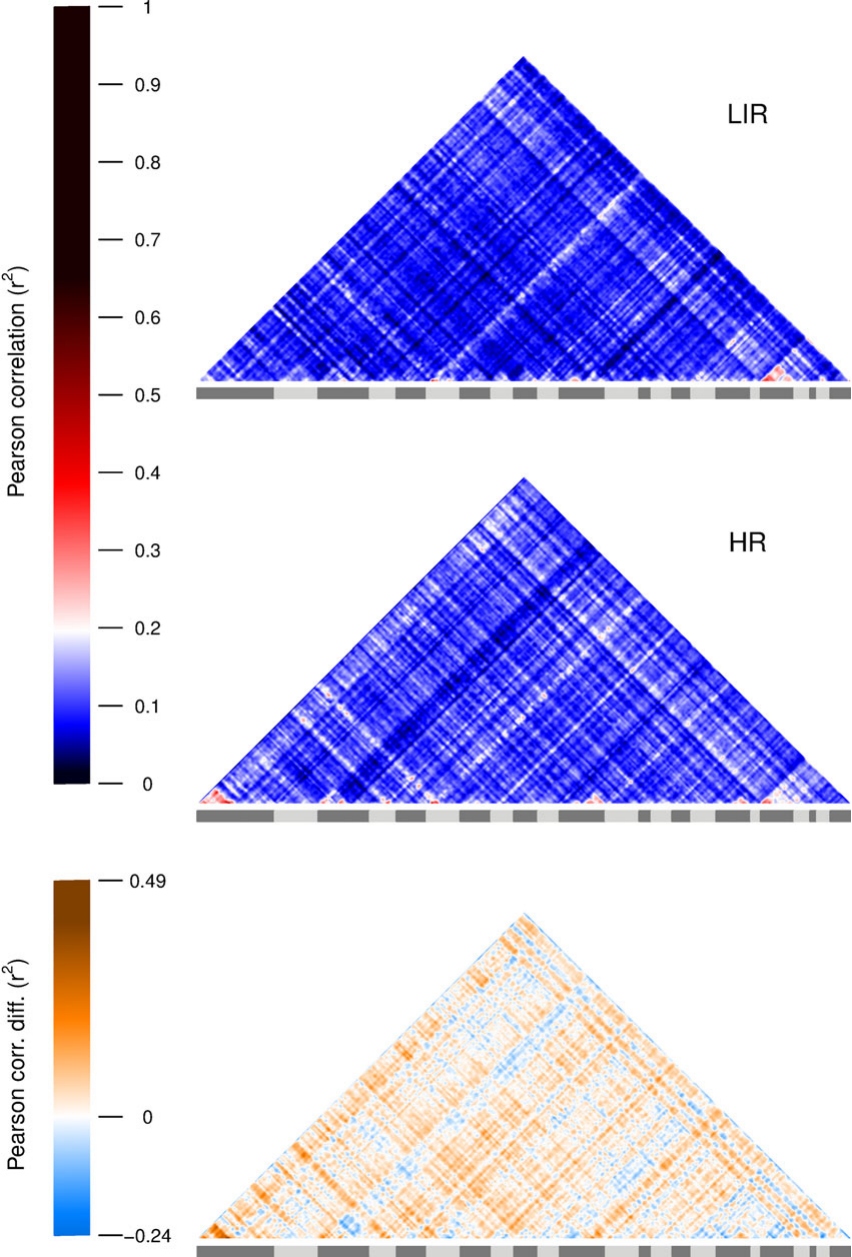
Genome‐wide correlation heatmap. Triangular heatmaps showing the Pearson correlations among gene expression values across the entire genome. Genes are ordered from left to right by chromosome and position. Correlations are calculated for LIR (top panel) and HR samples (middle panel), respectively. High correlation values (dark red) characterize blocks of neighboring genes emerging as small triangular‐shaped domains at the bottom of the heatmaps. Higher‐order triangular‐shaped patterns are also visible, identifying larger fields within which the average correlations are higher compared to longer‐range interactions. Blue values represent low correlations, characterizing insulation regions separating high‐correlation blocks. The bottom heatmap represents the arithmetic difference between the HR and the LIR heatmaps (HR minus LIR); positive values (orange) identify blocks in which the expression correlation is higher in HR samples compared to LIR samples. Negative values (cyan) show regions of higher interaction in LIR compared to HR ones.

When we mapped the TIN index values for each gene in the genome, we noticed a nonrandom distribution of clusters of contiguous genes showing similar TIN indexes (Fig. S5). The resulting discrete clusters with high TIN indexes are separated by regions with lower TIN index values, indicating the presence of domains with contiguous genes showing coordinated expressions. We also calculated the absolute Pearson correlation of the expression values of all gene pairs across all samples. After ordering genes by chromosome and position, the correlation matrix revealed that higher correlations preferentially involve pairs of neighboring genes, confirming the presence of CEDs separated by regions of low correlation giving rise to ‘plaid patterns’ (Sexton *et al*., 2012; de Wit and de Laat, 2012) (red and blue intersperse regions near the base of the triangular matrix, Fig. 4). Long‐distance intra‐ and interchromosomal correlations also emerged (upper regions in the triangular matrix, Fig. 4). When the expression correlation data are calculated within clinically meaningful subsets, such as risk groups, a more detailed and interesting picture emerges with group‐specific patterns of CEDs and insulator regions (Fig. 4). The mean of intrachromosome gene pair expression correlations decreases with the distance separating two genes (Fig. S6). The higher correlations, those that define the CEDs, are confined to sub‐megabase distances.

We also compared the expression distribution of single genes between LIR and HR samples and noticed that genes belonging to the CEDs that better discriminated the two groups of patients (Fig. S2 and Table S10) had good discriminatory potential on their own. Among these, the genes belonging to the TIN‐signature showed the greatest discerning capacity (Fig. S7).

These observations suggest that a possible functional relationship may underlie these gene connections at both local and long‐range distances.

### 4.3 Colocalizing genomic features

Hi‐C experiments, a technique instrumental in revealing the megabase‐level substructure of chromosomes, also called ‘topologically associating domains’ (TAD), facilitated the unveiling of an evolutionarily conserved connection among subchromosome structures, functional gene regulation, and genome instability (Ciabrelli and Cavalli, 2015; Dixon *et al*., 2012).

Topologically associating domains are chromatin domains hundreds of kbs in length that are characterized by a preferential physical self‐interaction of the intervening sequences. The linking of TADs to genome instability also shed light on mechanisms underlying some of the general features shared by most cancer cells (Mortusewicz *et al*., 2013; Wilson *et al*., 2015).

**Figure 5:**
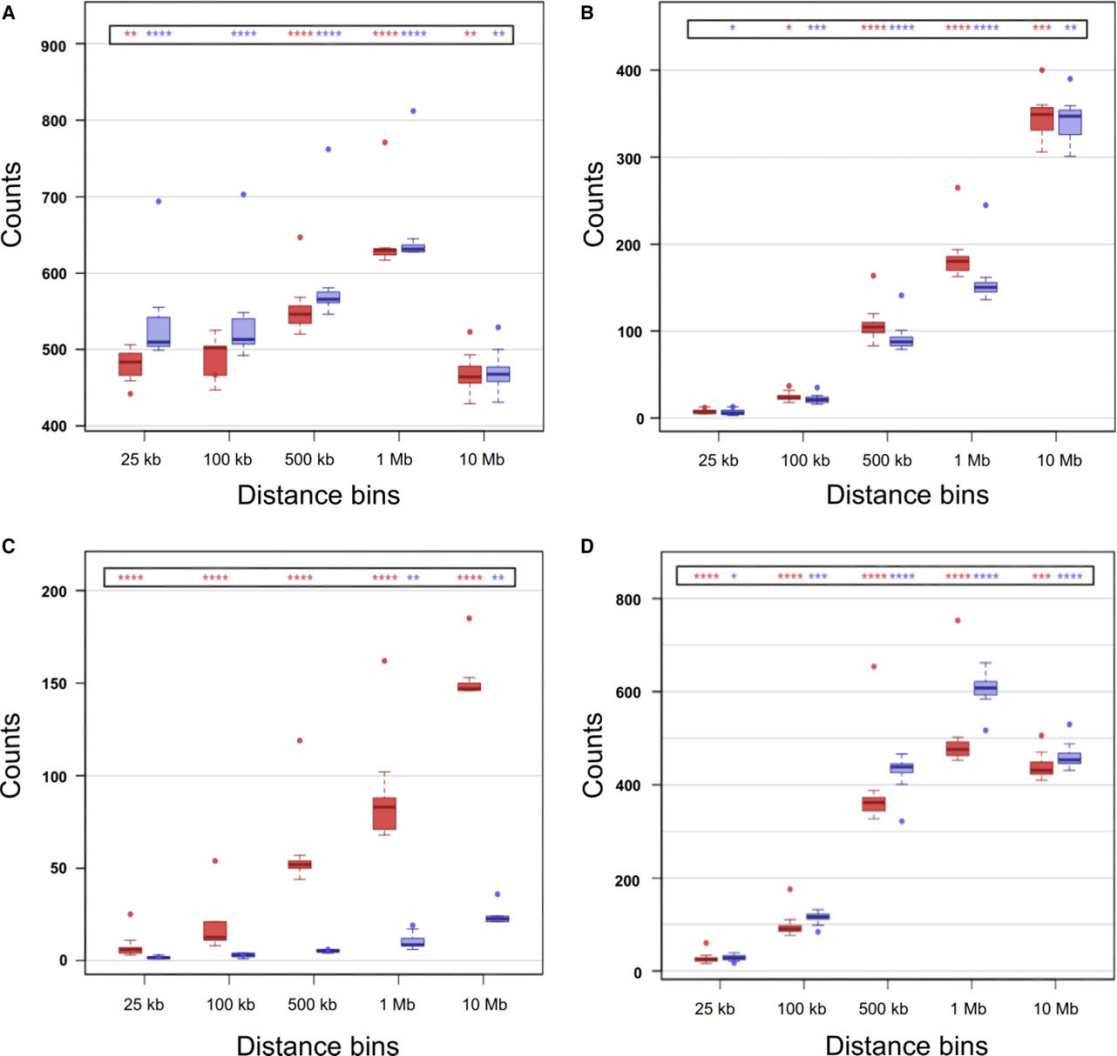
Colocalization between TIN‐signature genes and chromatin features. Colocalizations are defined as the co‐occurrence of two features within a given distance. (A) Counts of observed colocalizations of TIN‐signature genes with CED cores (the central regions of CEDs; red dots) and CED edges (the terminal edges of CEDs; blue dots) as a function of the distance between them. The boxplots represent the distributions of expected colocalization counts for CED cores (red) and CED edges (blue) at various distances. Asterisks on top of the boxplots show statistically significant deviations from expected to observed colocalizations: *P*‐values *5 × 10^−2^, **1 × 10^−2^, ***1 × 10^−3^, and ****1 × 10^−6^. For distances up to 500 kb, the colocalization between TIN‐signature genes and CED cores is not significant, whereas their colocalizations with CED edges are markedly significant, indicating that TIN‐signature genes are preferentially located at CED edges. (B) Colocalizations of TIN‐signature genes with CTCF (red) and cohesin complexes (blue dots). The colocalization between TIN‐signature genes and both CTCF and cohesin complexes is highly significant for distances ranging between 500 kb and 1.0 Mb, but not outside this range. (C) Colocalizations of TIN‐signature genes with H3K27ac peaks (marker of open‐active chromatin; red) and H3K4me1 (marker of condensed inactive chromatin; blue). At any distance, the colocalization between TIN‐signature genes and SE (H3K27ac) is highly significant; conversely, there is no significant colocalization of TIN‐signature genes with markers of compacted inactive chromatin (H3K4me1). (D) Colocalizations of TIN‐signature genes with early (red)‐ and late (blue)‐replicating genes. The colocalization between TIN‐signature genes and early‐replicating genes is highly significant at all distance ranges; conversely, the lack of TIN‐signature genes colocalizing (observed counts lower than expected) with late‐replicating genes becomes highly significant for distances between 100 kb and 1.0 Mb.

With this in mind, we tested whether the expression domains along with the TIN‐signature genes are ascribable to any of these structures using positional association with known genomic features. The positions of TIN‐signature genes were set as targets, and genomic features as queries with the purpose of testing for their physical colocalization. We ascertained the positional correlation between the targets and queries by counting the occurrences of overlaps between the elements of the two series and then compared the results to the number of intersections after randomly shuffling the positions of the queries. We first evaluated colocalizations between the CEDs and the TIN‐signature genes. Given that the CEDs are domains separated by very short boundaries, we defined their central region as CED cores and the remaining terminal parts as CED edges, and we then checked for colocalization between these two portions of the CEDs and the TIN‐signature genes. The results show a marked bias for the concurrence of TIN‐signature genes and CED edges (Fig. 5A). To further explore the positional association of the TIN‐signature genes with transcriptionally active loops, we focused our attention on chromatin structures associated with loops such as CTCF/cohesin binding domains (Tang *et al*., 2015). We observed a clear distance relationship between the positions of TIN‐signature genes and CTCF/cohesin binding sites (Fig. 5B). This relationship reaches a peak of statistical significance for colocalizations between 0.5 and 1.0 Mb, as would be expected in the case of a structural link between them in the context of active TADs. We then considered widespread regulatory elements like SEs, recently investigated for their altered activity in neuroblastoma, and their local effects on the expression of neighboring genes (Oldridge *et al*., 2015; Peifer *et al*., 2015; Valentijn *et al*., 2015). SEs were initially identified and characterized as regions with higher capability for transcription activity compared to typical enhancers (Hnisz *et al*., 2013). This increased potential for regulatory activity involves approximately 3% of the known enhancers in the genome (Pott and Lieb, 2014). SEs have been linked to the expression of key housekeeping genes and have been proposed as master regulators of tissue specificity determination and maintenance (Niederriter *et al*., 2015; Seton‐Rogers, 2014; Whyte *et al*., 2013). Their location has been associated with histone markers such as H3K4me1, HeK4me3, H3K27me3, and H3K27ac, with the latter being prominent. Indeed, clusters of strong and closely spaced acetylated H3K27 signals are considered to be good proxies for the presence of SEs and are well known to be associated with open and active chromatin. Overall, TIN‐signature genes show a highly significant (*P* < 10^−6^) enrichment for proximal SEs at all distance ranges measured (Fig. 5C). On the other hand, SE intersection with markers of compacted inactive chromatin, such as the monomethylation of H3K4, shows no significant association up to 1.0 Mb where it becomes marginally significant (Fig. 5C). Finally, we explored CED interactions with higher‐order chromatin structures like TADs. As replication timing has been coupled to TADs (Pope *et al*., 2014), we examined available data on replication timing calculated for the SK‐N‐SH neuroblastoma cell line. We detected colocalizations of CEDs with early‐replicating regions of the genome spanning distances within 1.0 Mb (Fig. 5D). This observation fits well with the finding that early replication domains are generally accessible and transcriptionally more active (Rivera‐Mulia *et al*., 2015; Wilson *et al*., 2015). Furthermore, early‐replicating regions could harbor conflicts between replication and transcription activities resulting in an increase in stalled/collapsed replication forks usually resolved through double‐strand break repair mechanisms. One of the consequences of these repairing processes frequently occurring within the same region is the incidence of recurrent rearrangements giving rise to fragile sites (Fungtammasan *et al*., 2012; Georgakilas *et al*., 2014). As for CEDs, genes of the TIN‐signature are preferentially located within TADs transition regions and replicated early during the S phase, where copy number variation‐related chromosomal breaks tend to cluster (Debatisse *et al*., 2012; Donley and Thayer, 2013; Yaffe *et al*., 2010). Taken together, these results unfold a strong connection between TIN‐signature genes and the proximity of functionally relevant regulatory elements like SE.

## 5 Discussion

Although genome instability, as a hallmark of cancer, is primarily exemplified by the well‐studied chromosomal instability as its main paradigm, very little is known about its counterpart: TIN. The aim of the present work was the systematic investigation of TIN as a genome‐wide phenomenon related to cancer. In neuroblastoma, pediatric cancer, both the localized and the metastatic disease are mainly characterized by the presence of genome instability‐associated chromosome alterations rather than point mutations. Neuroblastoma tumors typically have numerical chromosome changes at low stages and structural changes at high stages. In the present work, we examined the TIN using a publicly available large cohort of neuroblastoma samples belonging to patients of all clinical stages. We aimed to define and measure TIN and to explore its correlations with clinical and genomic features. This led to the observation that neuroblastomas in HR patients have elevated transcription variability compared to tumors from LIR patients. To our knowledge, this is the first systematic effort aimed at measuring and studying the TIN of neuroblastoma tumors as an aspect of cancer instability.

The results we report show that global transcriptional alteration is a good predictor of neuroblastoma patient outcome. In particular, high TIN levels are significantly associated with poor prognosis. The expression misregulation concerns not only a limited number of functionally relevant transcription modules but also a wider, seemingly stochastic, component involving a large part of the transcriptome. This global fluctuation of gene expression perturbs the tight transcriptional control of the normal ‘regulome’ (Buenrostro *et al*., 2015) of healthy cells, increasing their instability with unpredictable consequences. Evidence that this deteriorating regulation of transcription has structural origins is supported by the finding that the affected genes tend to physically cluster along the genome in domains of coordinated expression, mirroring the TAD substructure of the chromatin. This suggests a role for pre‐existing structural or epigenetic alterations of chromatin domains in fostering a generalized transcriptional deregulation, which eventually leads to a natural selection for the altered expression of cancer‐related transcripts. Indeed, we found a preferential misexpression of genes within domains harboring regulatory elements such as SE specifically active in neuroblastoma cell lines. Recent studies (Flavahan *et al*., 2016; Johann *et al*., 2016) support the notion that the epigenetic landscape may provide crucial insights into our understanding of the molecular basis of some cancers, particularly pediatric cancers (Feinberg *et al*., 2016). Pediatric cancers are prominently characterized by high heterogeneity, at both the morphological and molecular levels, with low rates of recurrent somatic alterations and expression signatures of controversial efficacy. Part of the still missing elements for refining the molecular picture of pediatric cancers are therefore likely to be found at the chromosome/chromatin structural and regulatory levels.

## 6 Conclusion

The new approach to expression data analysis we are proposing can be useful for re‐evaluating transcription data in cancer cells. This can further implement structural/regulatory information routinely produced by the novel next‐generation sequencing approaches. In this respect, the potential benefit of gathering chromatin‐related regulatory information from the transcriptome should not be underestimated, especially in diseases for which the available amount of tumor tissue is often a limiting factor, hindering a multidimensional molecular characterization of samples.

Overall, our observations strongly suggest that TIN captures at the transcriptional level crucial regulatory information of the chromatin structure in neuroblastoma samples. This approach of gene expression analysis broadens the perspective of genome instability investigations and contributes to bridging the gap between its structural and functional aspects.

## Author contributions

GPT jointly conceived and designed the study with CZ. CZ assembled input data, wrote codes, developed analytical tools, designed and implemented the stochastic simulation model, analyzed and interpreted data, wrote the manuscript and the Supporting Information. All authors discussed the results and implications and commented on the manuscript at all stages.

## Supporting information


**Fig. S1.** Data processing workflow.
**Fig. S2.** TIN‐index distribution.
**Fig. S3.** Unsupervised clustering of the ‘gene‐wise’ TIN‐index.
**Fig. S4.** Gene expression correlation as a function of inter‐gene distance.
**Fig. S5.** Examples of local correlation heatmaps.
**Fig. S6.** Examples of gene expression distribution in LIR and HR samples.
**Fig. S7.** Chromosome‐wide correlation heatmaps.
**Table S1.** Dataset prevalences of clinical features.
**Table S2.** Statistically significant results of the multivariate analysis.
**Table S3.** TIN‐signature genes.
**Table S4.** ROC curves test.
**Table S5.** Summary of the pathway enrichment analysis on the TIN‐signature genes.
**Table S6.** KEGG pathways enrichment analysis on the TIN‐signature.
**Table S7.** NCI pathways enrichment analysis on the TIN‐signature.
**Table S8.** Reactome pathways enrichment analysis on the TIN‐signature.
**Table S9.** Summary of the pathway enrichment analysis on the TIN‐signature genes, stratified by patients' clusters.
**Table S10.** Genes mapping within the intervals represented in the local correlation heatmaps of Fig. S5.
