# Supplementary material for: Transcription instability in high‐risk neuroblastoma is associated with a global perturbation of chromatin domains
Source: Mol Oncol. 2017 Oct 10;11(11):1646–58. doi: 10.1002/1878-0261.12139 (PMC5664000; doi:10.1002/1878-0261.12139)
Supplement: Supplementary file 1 — Fig. S1. Data processing workflow. Fig. S2. TIN‐index distribution. Fig. S3. Unsupervised clustering of the ‘gene‐wise’ TIN‐index. Fig. S4. Gene expression correlation as a function of inter‐gene distance. Fig. S5. Examples of local correlation heatmaps. Fig. S6. Examples of gene expression distribution in LIR and HR samples. Fig. S7. Chromosome‐wide correlation heatmaps. Table S1. Dataset prevalences of clinical features. Table S2. Statistically significant results of the multivariate analysis. Table S3. TIN‐signature genes. Table S4. ROC curves test. Table S5. Summary of the pathway enrichment analysis on the TIN‐signature genes. Table S6. KEGG pathways enrichment analysis on the TIN‐signature. Table S7. NCI pathways enrichment analysis on the TIN‐signature. Table S8. Reactome pathways enrichment analysis on the TIN‐signature. Table S9. Summary of the pathway enrichment analysis on the TIN‐signature genes, stratified by patients' clusters. Table S10. Genes mapping within the intervals represented in the local correlation heatmaps of Fig. S5. [file MOL2-11-1646-s001.pdf]

Transcription instability in high-risk neuroblastoma is associated with a global perturbation of chromatin domains

Carlo Zanon[1] and Gian Paolo Tonini[1]

[1]Neuroblastoma Laboratory, Pediatric Research Institute, Citta' della Speranza, 35127 Padua, Italy

Corresponding author: Carlo Zanon

E-mail: c.zanon@irpcds.org

Phone: +39 0499640124

Running title: “Transcription instability in neuroblastoma”

Keywords: transcriptional instability, super-enhancer, chromatin structural domain

Abbreviations:

- HR, high-risk
- LIR, low/intermediate risk
- SE, super-enhancers
- CED, coordinated expression domains
- TAD, topologically-associating domains

504 NB samples

(all stages: 1,2,3,4,4S; E-MTAB-161 dataset, ArrayExpress-EMBL)

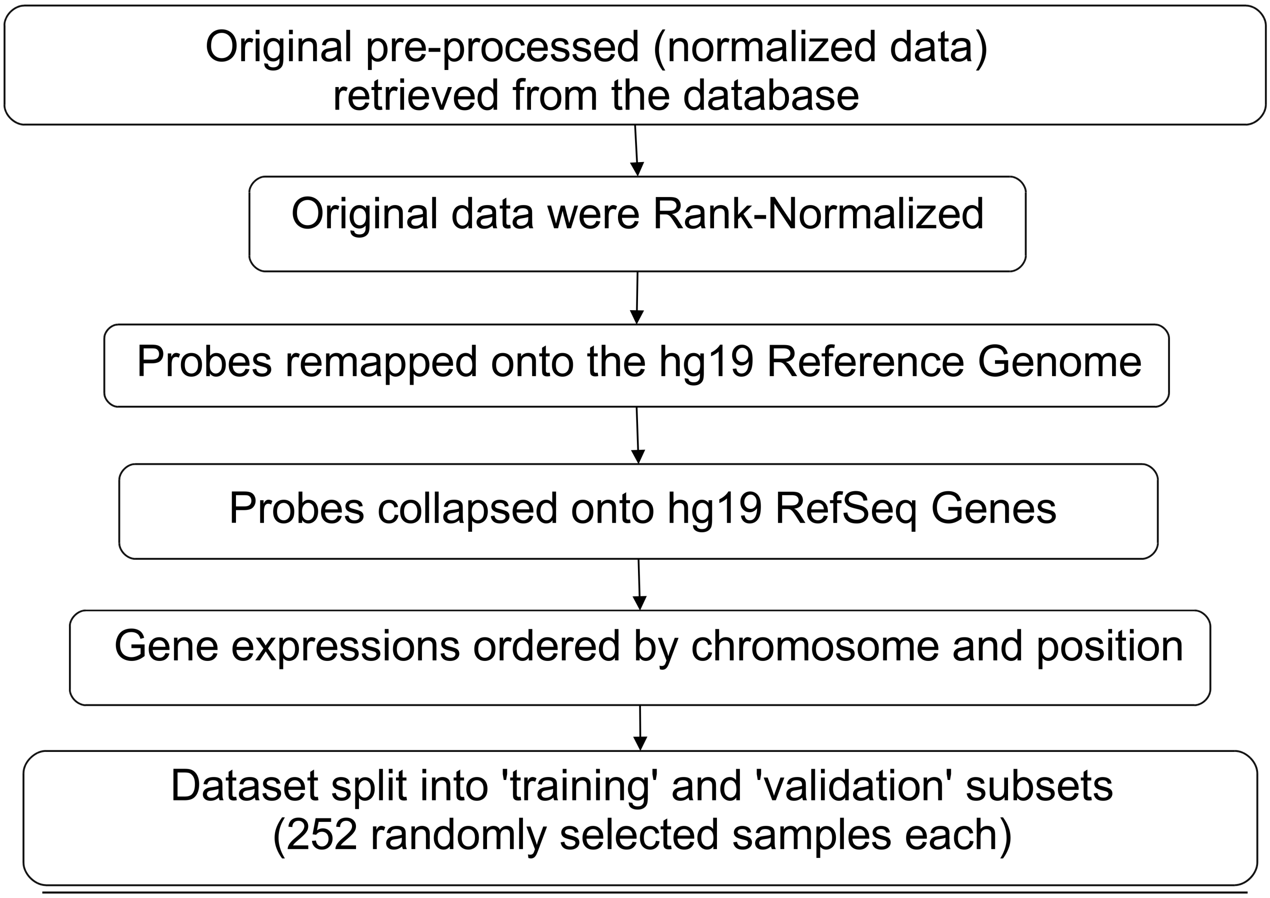

Supplementary Figure 1. Data processing workflow.

The original preprocessed data of the E-TAB-161 dataset retrieved from the ArrayExpress database and rank-normalized for each sample. Sequences of the probes are remapped onto the hg19 reference genome, and only the probes showing unambiguous mapping are retained for further analyses. Rank-normalized expression values were collapsed to hg19 RefSeq genes; the mean values of the probes were used in case of multiple mapping to one gene. Finally, gene expression values are ordered by chromosome and position to obtain a position-ordered gene expression data-matrix. Training and validation subsets were created by randomly assigning the 504 samples to each group.

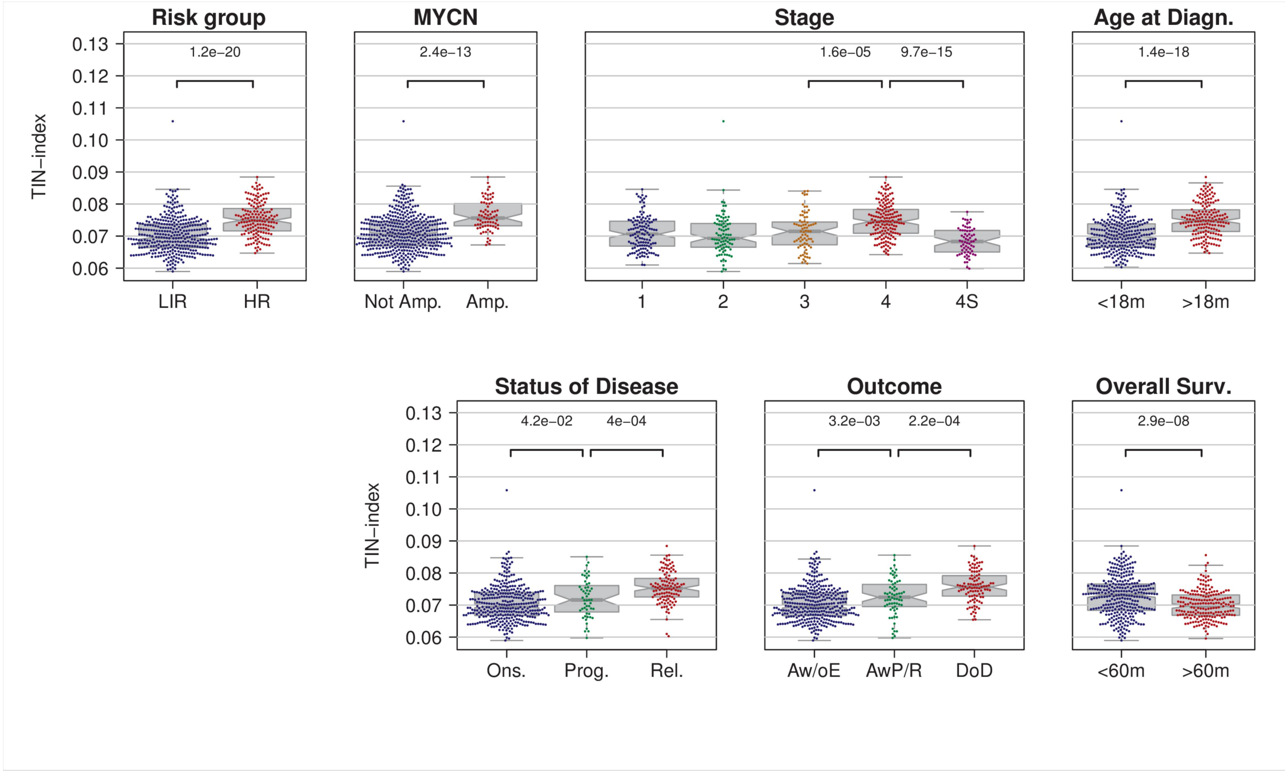

Supplementary Figure 2. TIN-index distribution.

Boxplots representing the distribution of TIN-indexes (calculated using the mean expression values across all 504 samples as the reference) stratified by relevant clinical features. Wilcox-test p-values of the statistically significant differences are reported on top. LIR=Low/Intermediate Risk; HR=High-Risk; NotAm.=Not Amplified; Am.=Amplified; Ons.=Onset; Prog.=Progression; Rel.=Relapse; Aw/oE=Alive without Event; AwP/R=Alive with Progression or Relapse; DoD=Dead of Disease; m=months.

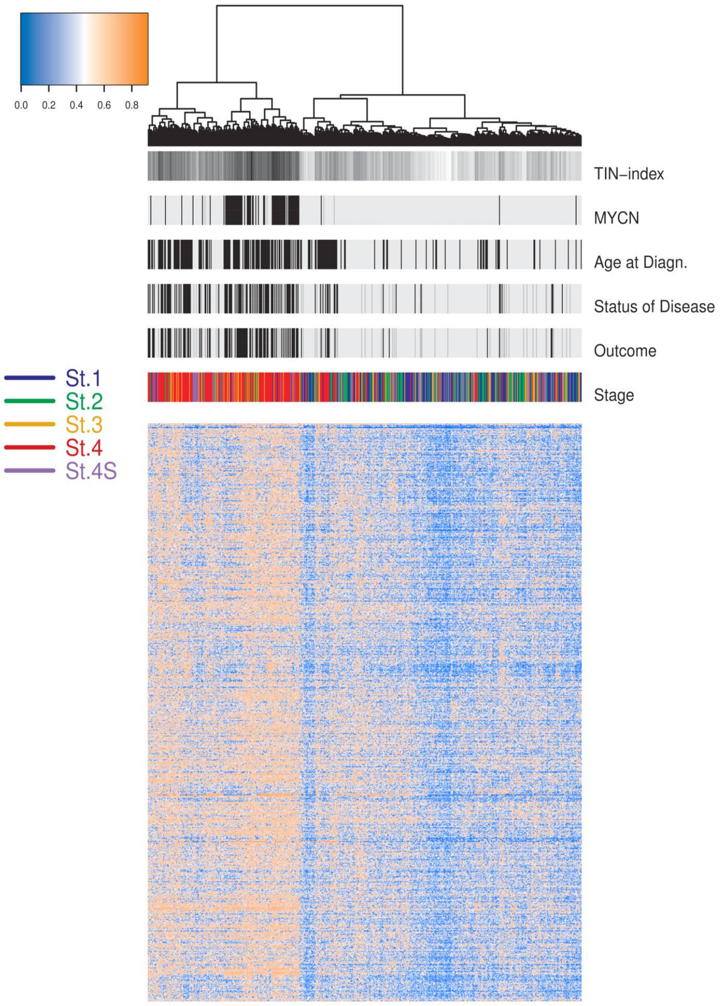

Supplementary Figure 3. Unsupervised clustering of the 'gene-wise' TIN-index.

Heatmap showing the unsupervised clustering of samples using the moving median of the 'gene-wise' TIN-index calculated over windows of 11 contiguous genes. The 'gene-wise' TIN-index corresponds to the squared deviation of each gene expression value from a reference value calculated as the mean expression value across the whole dataset. From bottom to top, the genes are ordered by chromosome and position. Relevant clinical features and TIN-indexes are reported on top; black bars represent unfavorable scores; light-grey bars represent favorable scores. The stage color code is shown in the legend.

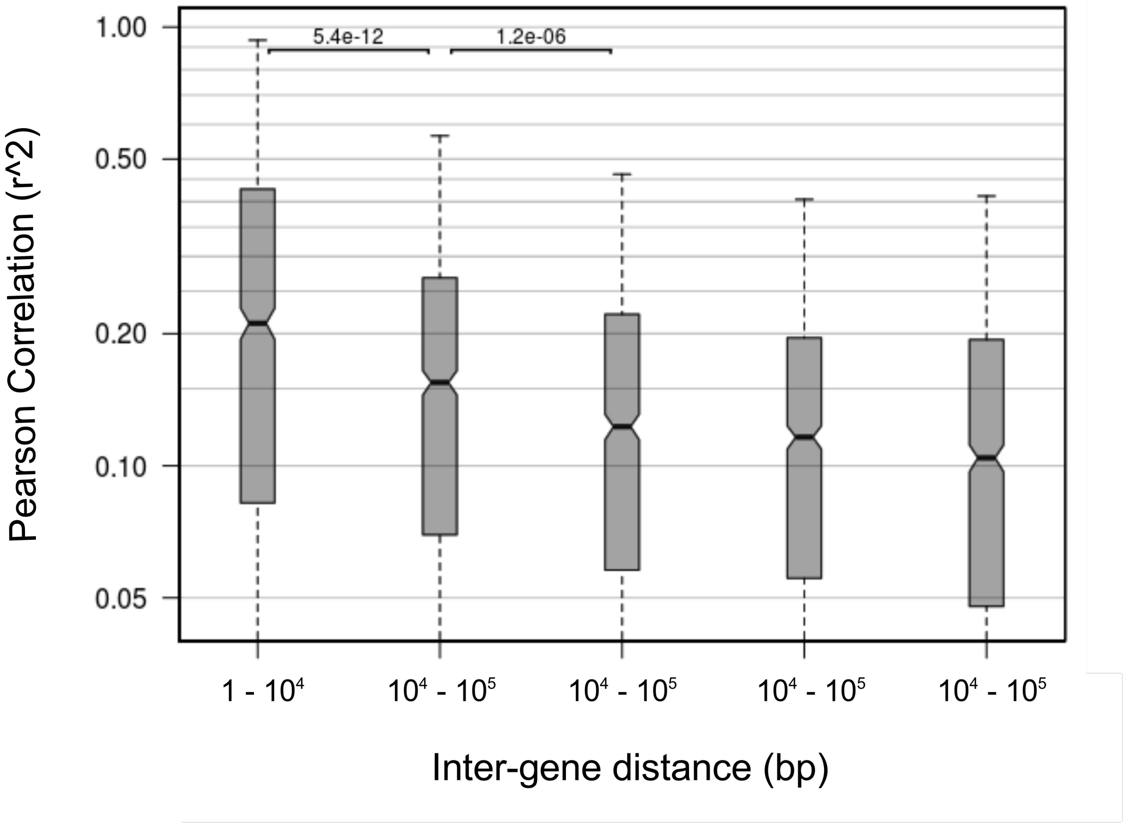

Supplementary Figure 4. Gene expression correlation as a function of inter-gene distance.

Boxplots showing the distribution of the Pearson correlation of gene expressions values between pairs of genes as a function of their inter-gene distance. On the X-axis are the inter-gene distance bins. As the distance increases, the median gene expression correlation decreases, as would be expected in the case of a positional effect in gene expression activity among neighboring genes. Numerical values on top represent the Wilconox test p-values of significant differences showing that the positional effect on gene expression correlation disappears as the intergenic distance rises above 1.0 Mb.

Supplementary Figure 5. Examples of local correlation heatmaps.

Triangular heatmaps with Pearson correlations among gene expressions values within localized regions spanning 50 genes. Genes are ordered from left to right by position along the chromosome. Correlations are calculated for LIR (top panel) and HR samples (middle panel). The bottom heatmap represents the arithmetic difference between the HR and the LIR heatmaps (HR minus LIR). Color codes are described in detail in the legend of Supplementary Figure 5. Arrows and the corresponding labels at the bottom of each panel mark the positions of known genes (in black) and genes belonging to the TIN-signature (in blue). Regions known to harbor chromosomal alterations in neuroblastoma are reported in panels a-e, conversely regions with no recurrent chromosomal alterations are shown in panels f-h. a) p-term region of chromosome 1; b-c) p-arm region of chromosome 2; d) p-term region of chromosome 5; e-f) p-arm and q-arm regions respectively of chromosome 17; g) q-arm region of chromosome 10; h) q-arm region of chromosome 11.

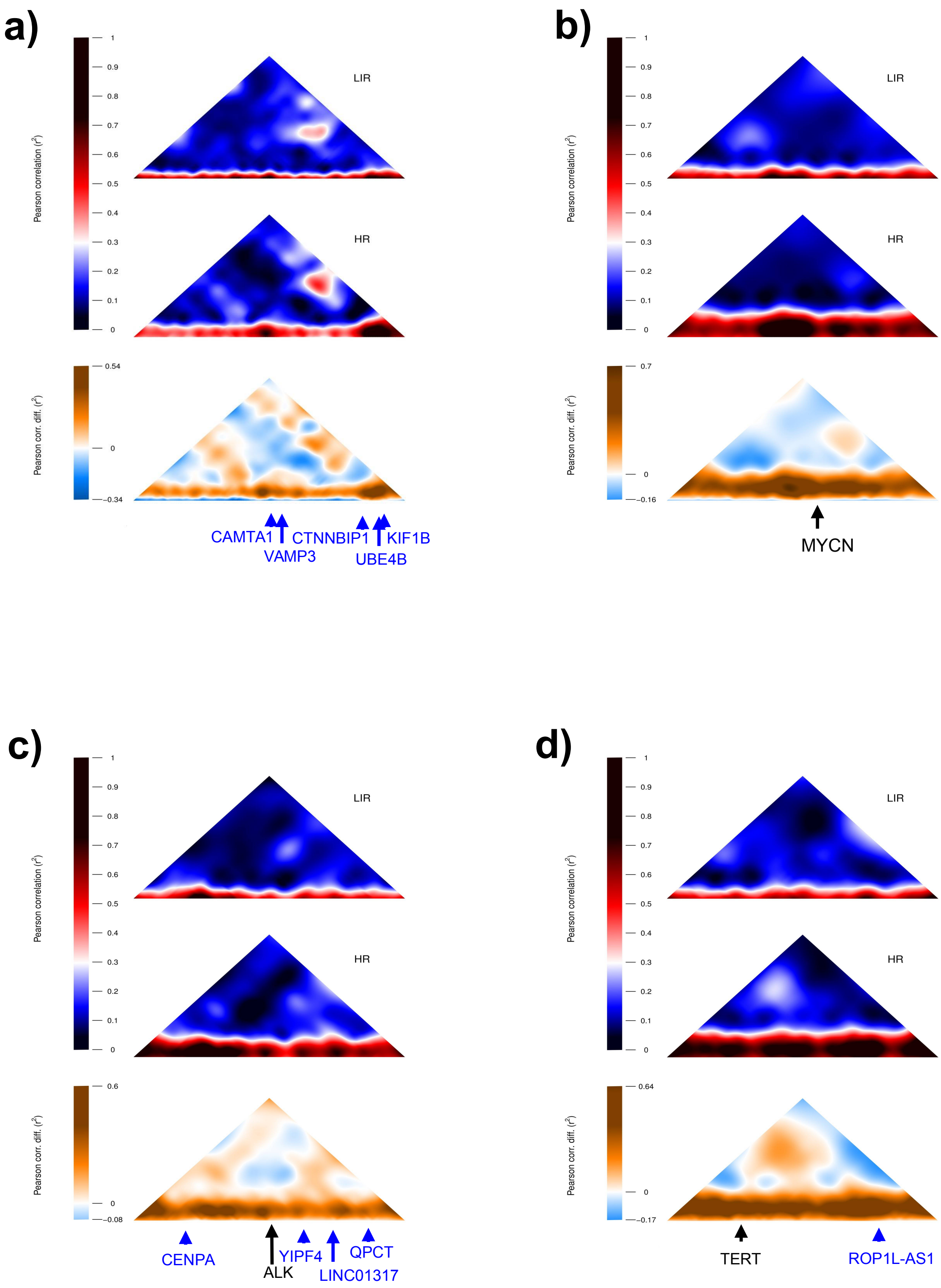

Supplementary Figure 5. Local correlation heatmaps (regions in chr1, chr2 and chr5).

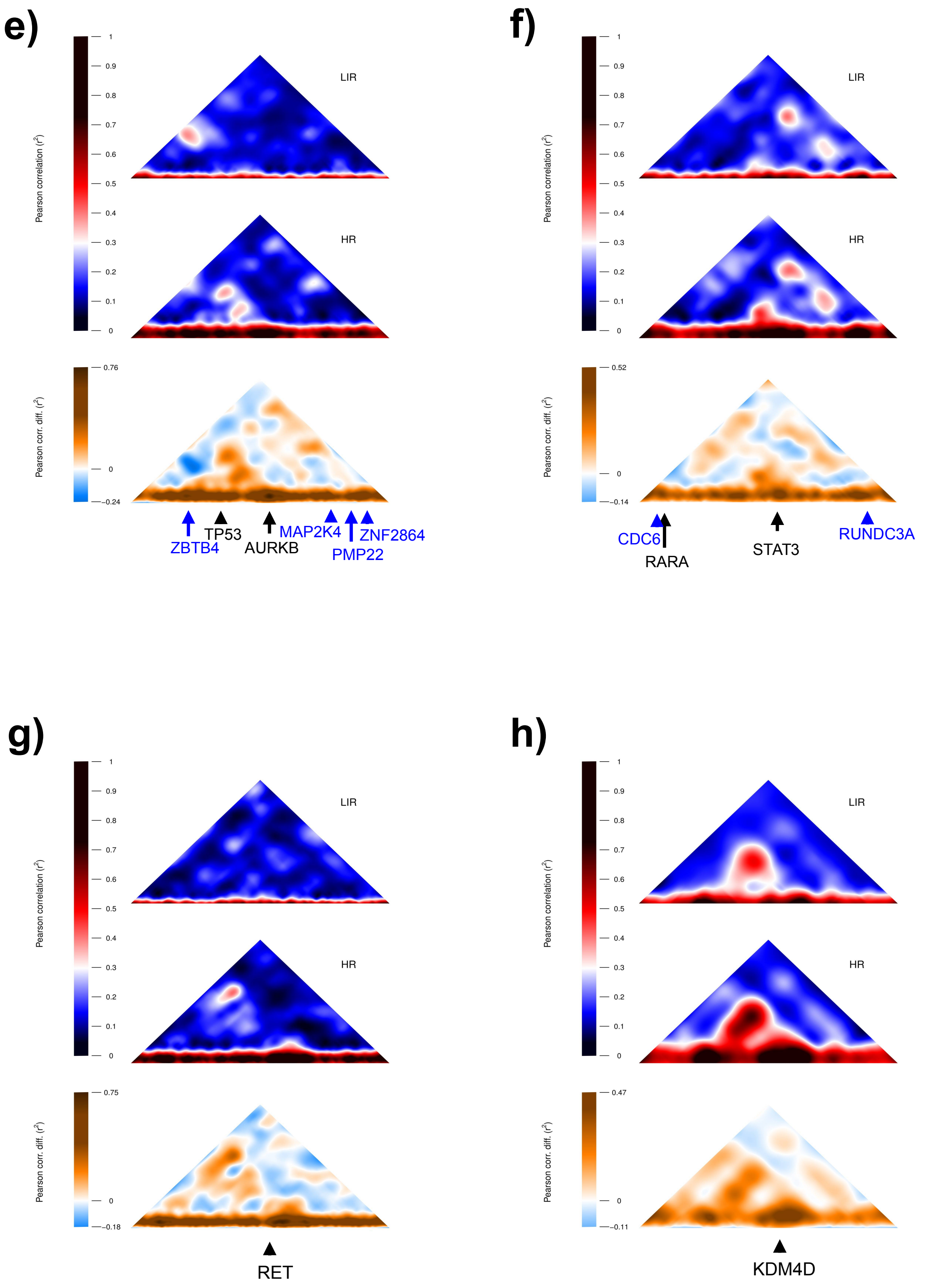

Supplementary Figure 5. Local correlation heatmaps (regions in chr17, chr10 and chr11).

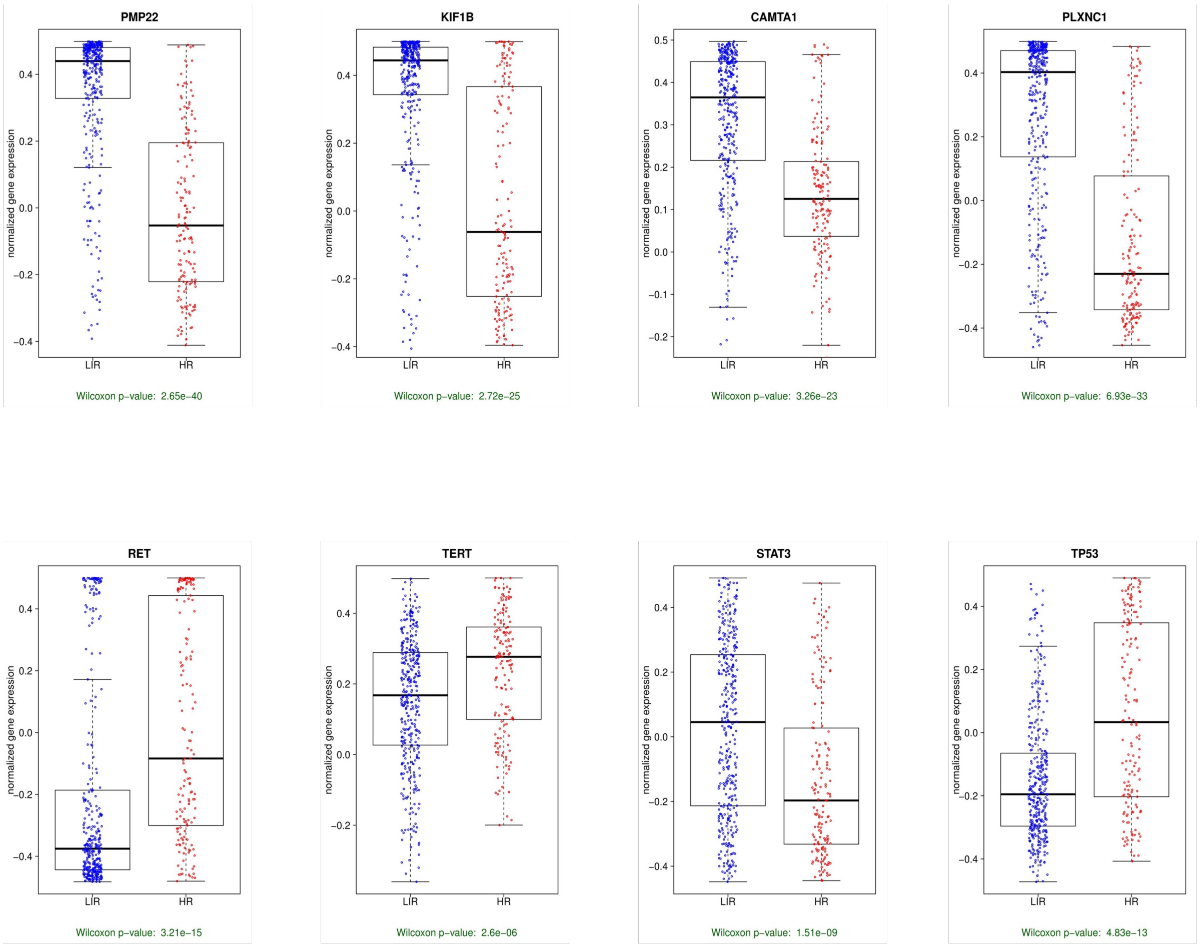

Supplementary Figure 6. Examples of gene expression distribution in LIR and HR samples.

Genes were selected among the TIN-signature genes (top row: PMP22, KIF1B, CAMTA1, PLXNC1) and among genes within prominent CEDs (bottom row: RET, TERT, STAT3, TP53). At the bottom of each panel the Wilcoxon p-value for the difference between the groups is reported. The p-values of TIN-signature genes (range 3.26×10^{-23}-2.65×10^{-40}) are more significant than those of genes within prominent CEDs (range 2.6×10^{-6}-3.21×10^{-15}) , indicating that they have greater discriminating power in separating LIR from HR samples.

Supplementary Figure 7. Chromosome-wide correlation heatmaps.

Triangular heatmaps showing Pearson correlations among gene expressions values across single chromosomes and for each chromosome. Genes are ordered from left to right by position: p-arm (left) and q-arm (right). Correlations are calculated for LIR (top panel) and HR samples (middle panel). High correlation values (dark-red) characterize blocks of neighboring genes emerging as small triangular-shaped domains at the bottom of the heatmap. Higher order triangular-shaped patterns are also visible, identifying larger fields within which the average correlations are higher than longer-range interactions. Blue values represent low correlations, characterizing insulation regions separating the higher-correlation blocks. The bottom heatmap represents the arithmetic difference between the HR and the LIR heatmaps (HR minus LIR); positive values (orange) identify blocks in which the expression correlation is higher in HR samples than LIR samples, and negative values (cyan) show regions of higher interaction in LIR compared to HR ones. Different chromosomes show different profiles of expression correlation. Some chromosome regions show larger differences between the risk groups and correspond to chromosome (see: 1p, 11q, 17q) domains whose CNV profiles are frequently altered in HR tumors.

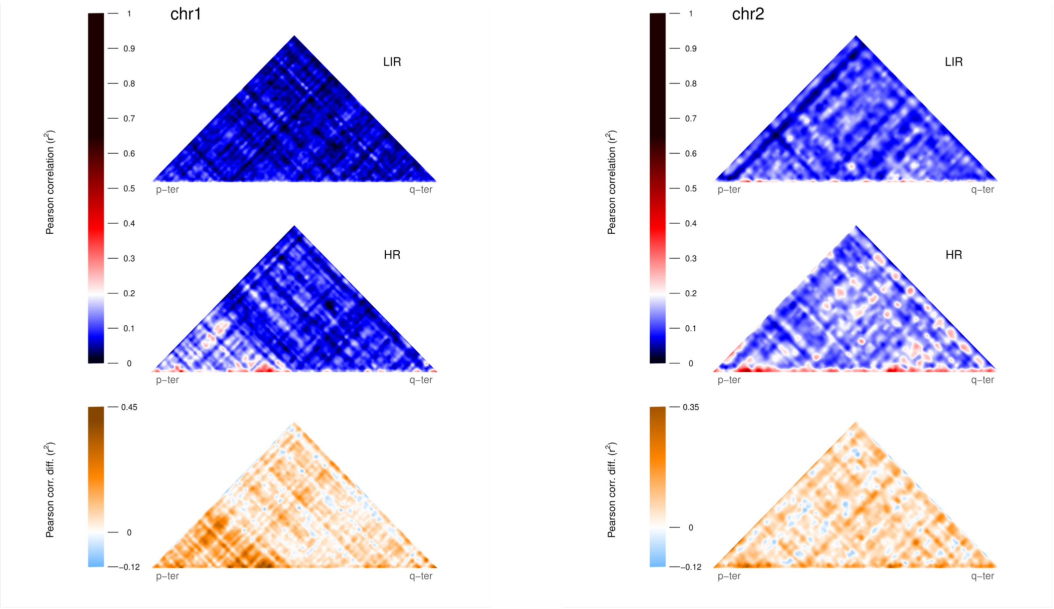

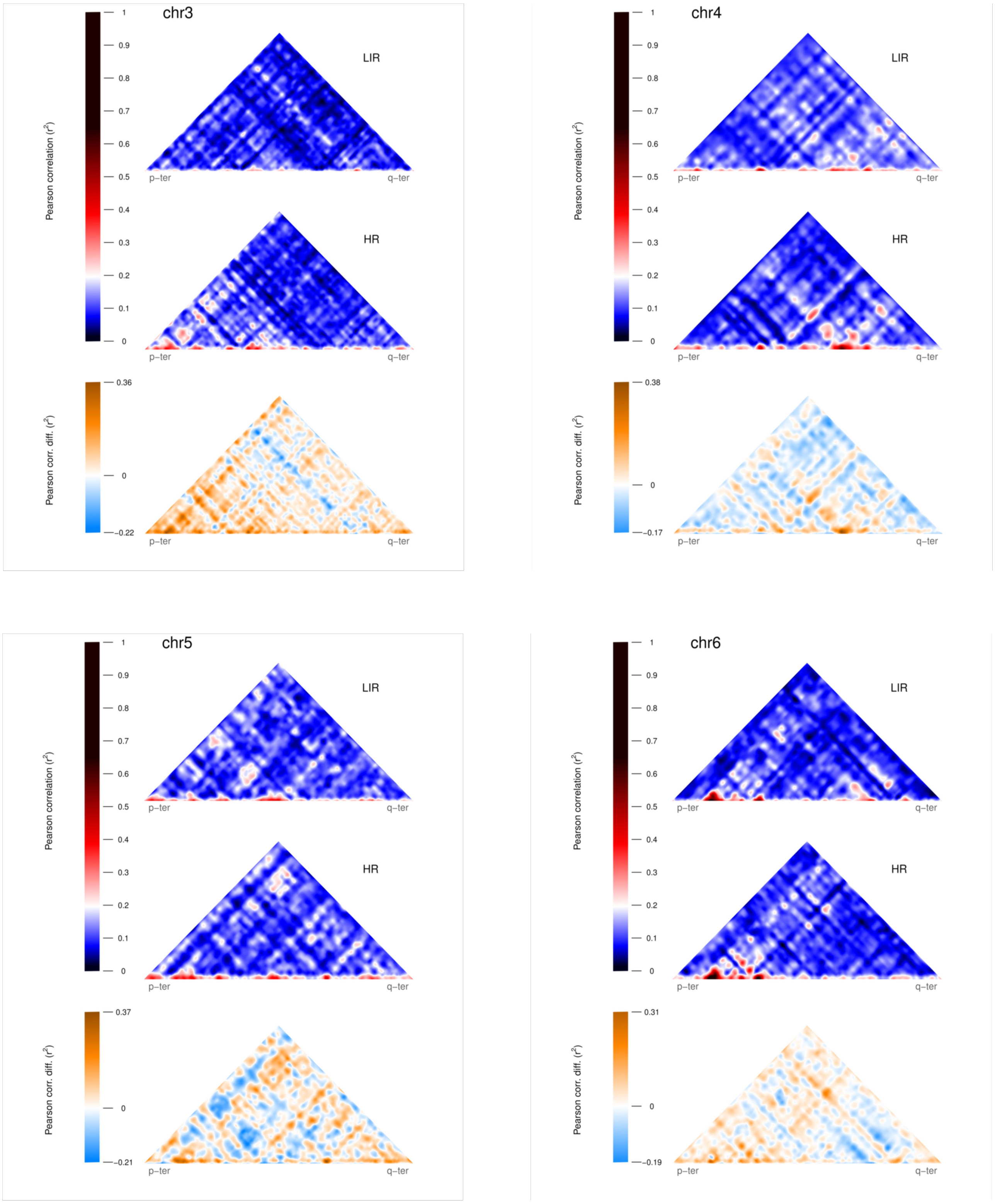

Supplementary Figure 7. Chromosome-wide correlation heatmaps (chr3, chr4, chr5 and chr6).

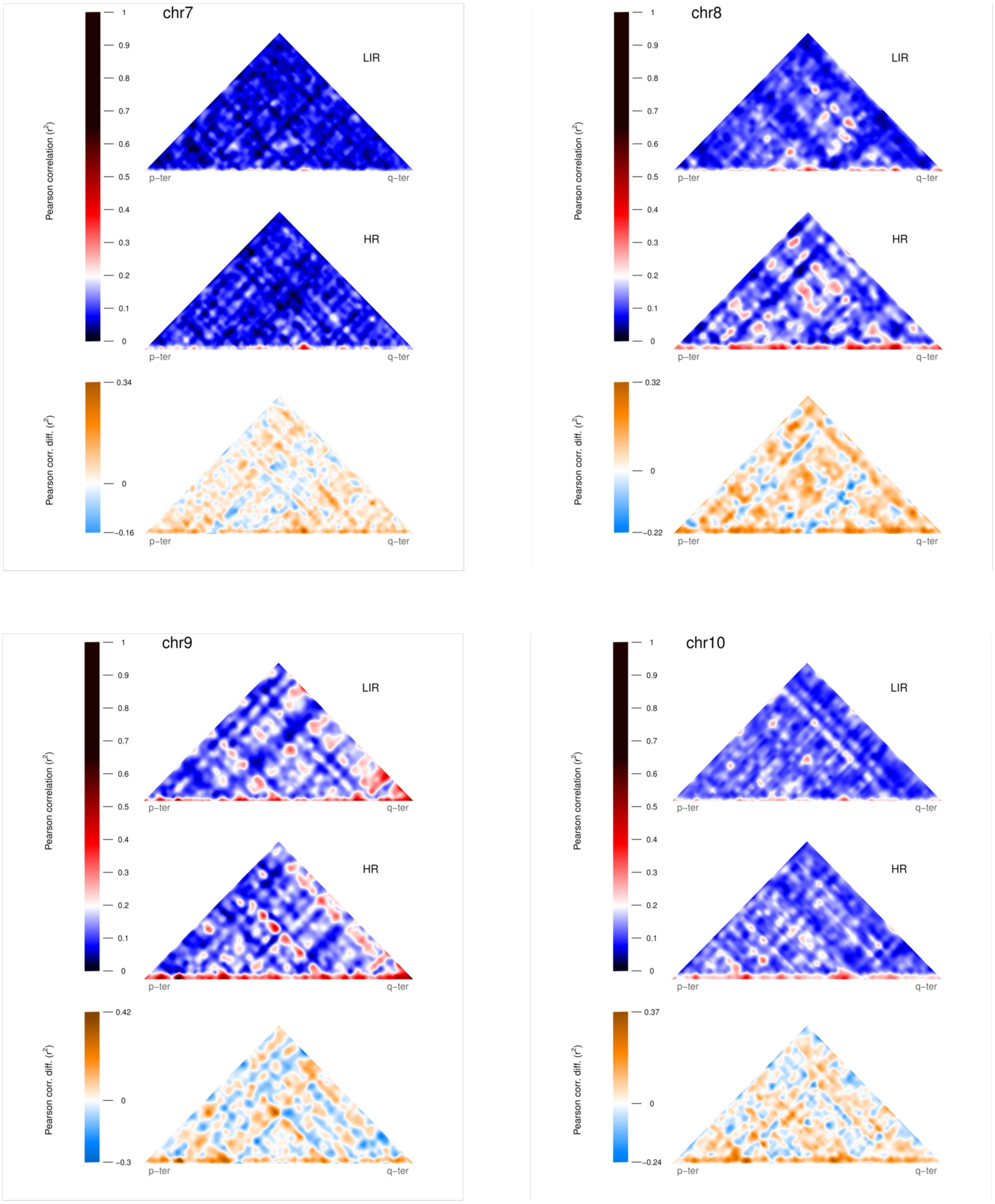

Supplementary Figure 7. Chromosome-wide correlation heatmaps (chr7, chr8, chr9 and chr10).

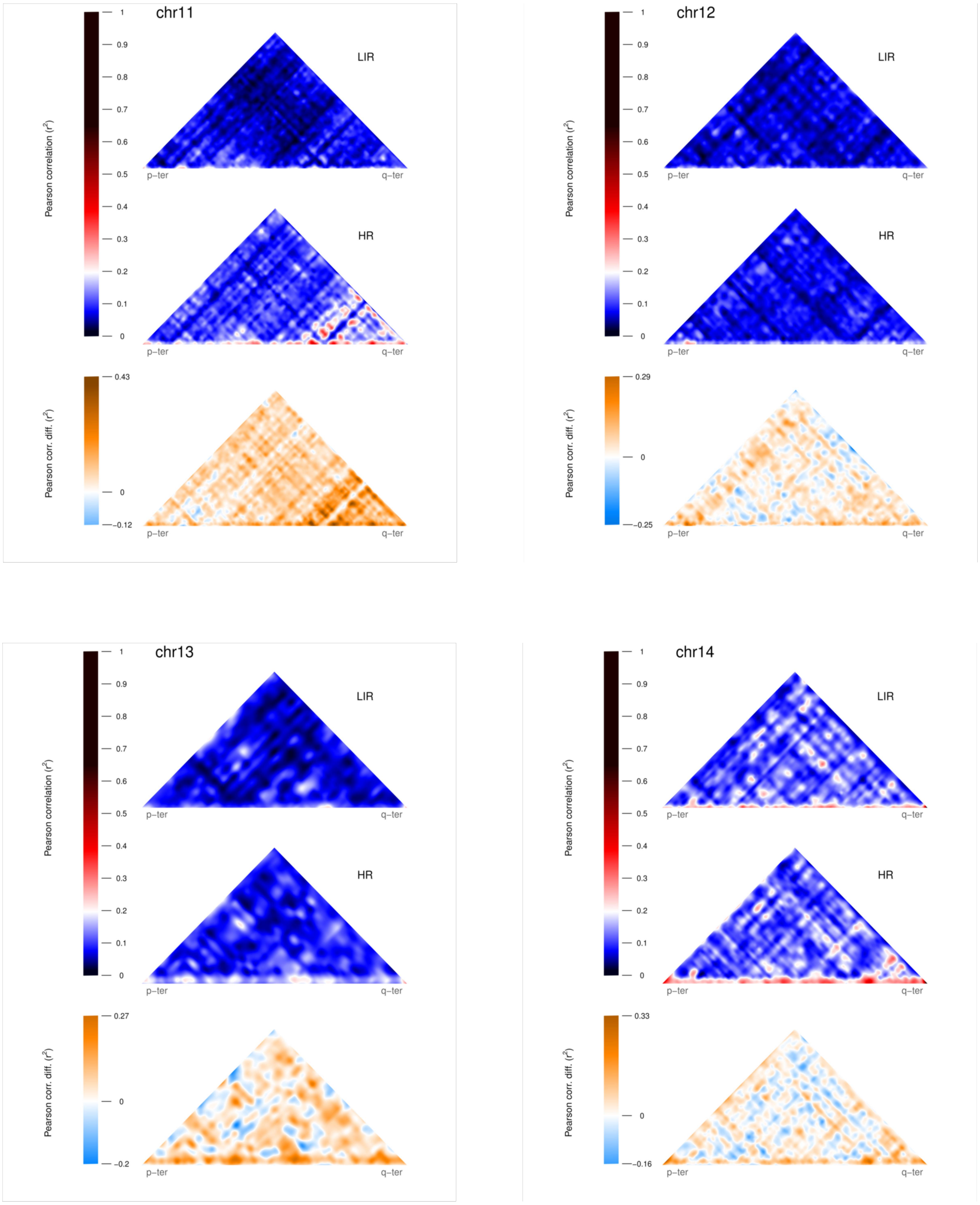

Supplementary Figure 7. Chromosome-wide correlation heatmaps (chr11, chr12, chr13 and chr14).

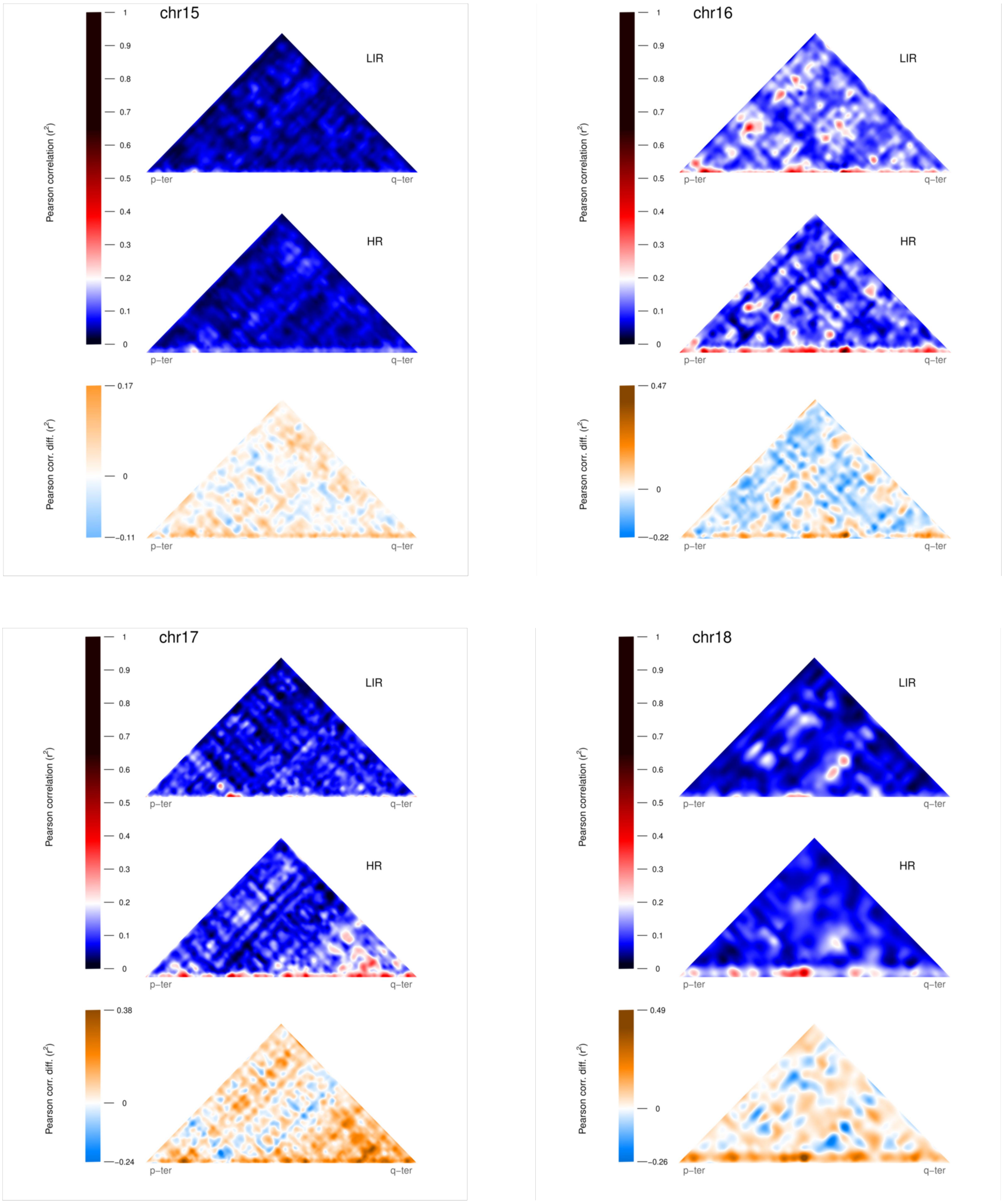

Supplementary Figure 7. Chromosome-wide correlation heatmaps (chr15, chr16, chr17 and chr18).

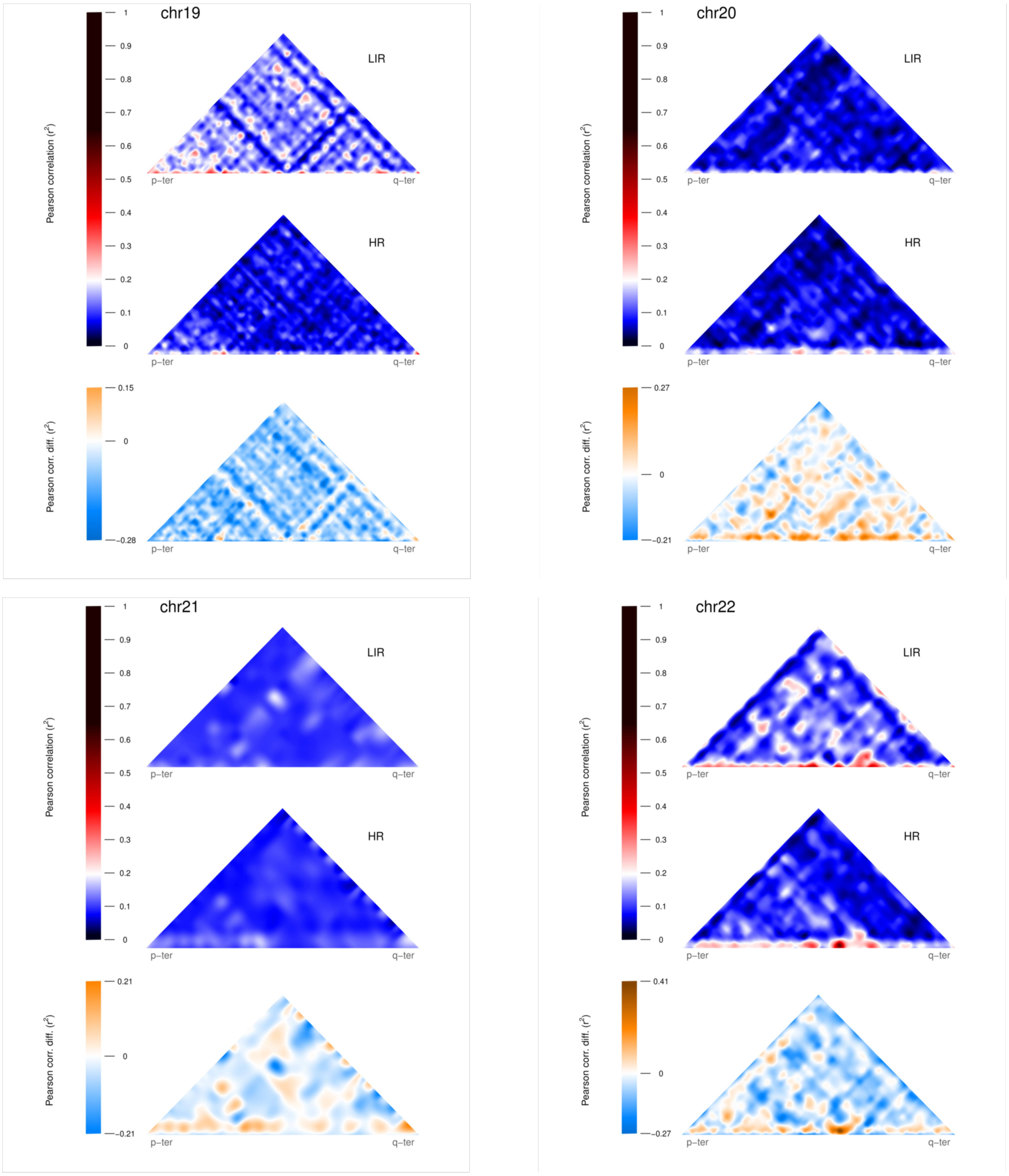

Supplementary Figure 7. Chromosome-wide correlation heatmaps (chr19, chr20, chr21 and chr22).

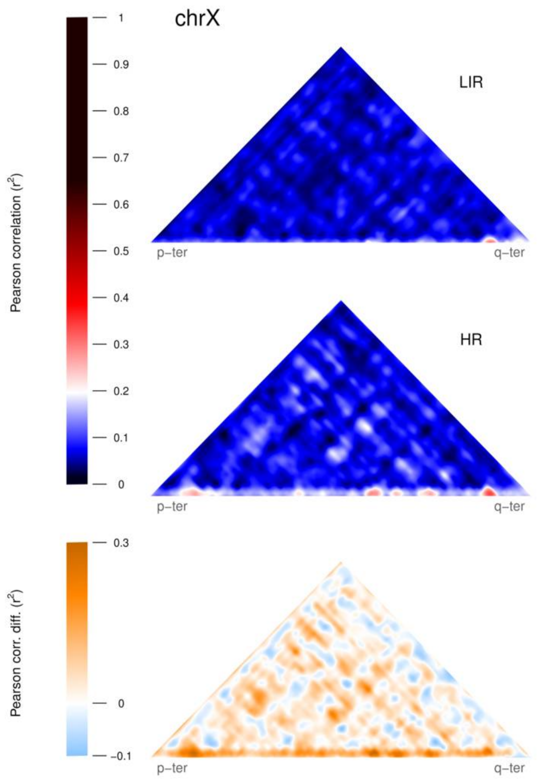

Supplementary Figure 7. Chromosome-wide correlation heatmaps (chrX).

	group	frequency	(%)
Stage:	stage-1	124	25%
	stage-2	86	17%
	stage-3	73	14%
	stage-4	156	31%
	stage-4S	65	13%
Age at Diagnosis:	younger than 18 months	282	62%
	older than 18 months	171	38%
MYCN:	Amplified	71	14%
	Not-amplified	427	85%
	MYCN-gain	5	1%
Risk Group:	Low-Intermediate	350	69%
	High	154	31%

Supplementary Table 1. Dataset prevalences of clinical features.

The distribution of samples among the subgroups of the four most important prognostic factors for neuroblastoma were checked for accordance to their general prevalence in the population (see Online Methods for details). First column reports the four prognostic factors; second column the subgroups within each factor; the third column the frequency of samples within each subgroup and the last column the corresponding proportion of the total samples as a percentage.

	coef	exp(coef)	se(coef)	z	p-value
Age_at_Diagnosis	0.00971	1.00976	0.00195	4.97	0.00000067
MYCN_status (MYCN Amplified)	1.1182	3.05933	0.24975	4.48	0.0000076
TIN_index	74.6	2.530000E+032	19.7	3.79	0.00015
Stage (Stage 4)	2.09866	8.15525	0.81009	2.59	0.00958
Risk_Group (High Risk)	0.97817	2.65957	0.42383	2.31	0.021
Stage (Stage 3)	1.63907	5.15036	0.81025	2.02	0.04308

Supplementary Table 2. Statistically significant results of the multivariate analysis.

The table shows the prognostic factors in decreasing order of statistical significance for their hazard ratios (output of the 'coxph' function of R statistical software). The first column reports the significant prognostic factors, the second column the coefficients of the Cox regression, the third column the hazard ratio (i.e. the exponential of the coefficient of Cox regression), the fourth column the z-scores and the last column the p-values.

RefSeq_Accession	Chromosome	Start	End	Symbol	Description
NR_038934	chr1	6845383	6948261	CAMTA1	calmodulin binding transcription activator 1
NM_004781	chr1	7831328	7841492	VAMP3	vesicle-associated membrane protein 3
NM_020248	chr1	9908333	9970316	CTNNBIP1	beta-catenin-interacting protein 1
NM_006048	chr1	10093040	10241296	UBE4B	ubiquitin conjugation factor E4 B isoform 2
NM_183416	chr1	10270763	10368655	KIF1B	kinesin-like protein KIF1B isoform alpha
NM_002685	chr1	11126669	11159967	EXOSC10	exosome component 10 isoform 2
NM_018156	chr1	12290095	12572098	VPS13D	vacuolar protein sorting-associated protein 13D isoform 2
NM_001328124	chr1	17944807	18024364	ARHGEF10L	rho guanine nucleotide exchange factor 10-like protein isoform 5
NM_004091	chr1	23832919	23857712	E2F2	transcription factor E2F2
NM_177424	chr1	28099693	28150963	STX12	syntaxin-12
NM_012090	chr1	39549838	39952810	MACF1	microtubule-actin cross-linking factor 1
NM_199342	chr1	43272722	43283059	SVBP	small vasohibin-binding protein
NM_014372	chr1	51701944	51739119	RNF11	RING finger protein 11
NM_148907	chr1	52195485	52254891	OSBPL9	oxysterol-binding protein-related protein 9 isoform c
NM_017737	chr1	93913687	94020218	FNBP1L	formin-binding protein 1-like isoform 2
NM_001010883	chr1	109102970	109181949	FAM102B	protein FAM102B
NM_014969	chr1	109512837	109584850	WDR47	WD repeat-containing protein 47 isoform 2
NM_003051	chr1	113454469	113498975	SLC16A1	monocarboxylate transporter 1
NM_001346068	chr1	143904508	143913160	FAM72C	protein FAM72C isoform 3
NM_006818	chr1	151032150	151040973	MLLT11	protein AF1q
NM_014970	chr1	169890469	170043879	KIFAP3	kinesin-associated protein 3 isoform 1
NM_015569	chr1	171810617	172381857	DNM3	dynamin-3 isoform a
NM_018254	chr1	211433288	211489725	RCOR3	REST corepressor 3 isoform d
NM_016343	chr1	214776531	214837914	CENPF	centromere protein F
NR_001587	chr1	220439520	220441057	AURKAPS1	aurora kinase A pseudogene 1
NR_102347	chr1	224544512	224564586	CNIH4	cornichon family AMPA receptor auxiliary protein 4
NM_006027	chr1	242012032	242053241	EXO1	exonuclease 1 isoform b
NM_001042426	chr2	27008882	27017455	CENPA	histone H3-like centromeric protein A isoform b
NM_032312	chr2	32502957	32531658	YIPF4	protein YIPF4
NR_126403	chr2	33931952	34522813	LINC01317	long intergenic non-protein coding RNA 1317
NM_012413	chr2	37571752	37600465	QPCT	glutaminyl-peptide cyclotransferase precursor
NM_018084	chr2	55514977	55647057	CCDC88A	girdin isoform 2
NM_145644	chr2	86426555	86440477	MRPL35	39S ribosomal protein L35, mitochondrial isoform b
NM_198461	chr2	100889752	100939195	LONRF2	LON peptidase N-terminal domain and RING finger protein 2
NM_153836	chr2	101964815	102003965	CREG2	protein CREG2 precursor
NM_006609	chr2	128056244	128100805	MAP3K2	mitogen-activated protein kinase kinase kinase 2
NR_110586	chr2	160549706	160568946	BAZ2B	bromodomain adjacent to zinc finger domain 2B
NM_006922	chr2	165944029	166060577	SCN3A	sodium channel protein type 3 subunit alpha isoform 1
NM_001040143	chr2	166152282	166248820	SCN2A	sodium channel protein type 2 subunit alpha isoform 2
NR_110260	chr2	166938040	167158293	LOC101929680	uncharacterized LOC101929680
NR_045628	chr2	167260082	167343481	SCN7A	sodium voltage-gated channel alpha subunit 7
NM_001320884	chr2	172544181	172606668	DYNC1I2	cytoplasmic dynein 1 intermediate chain 2 isoform 4
NM_014905	chr2	191745546	191830270	GLS	glutaminase kidney isoform, mitochondrial isoform 1 precursor
NM_004044	chr2	216176678	216214496	ATIC	bifunctional purine biosynthesis protein PURH
NM_032726	chr2	219472487	219501909	PLCD4	1-phosphatidylinositol 4,5-bisphosphate phosphodiesterase delta-4
NM_003469	chr2	224461657	224467217	SCG2	secretogranin-2 precursor
NM_139072	chr2	230222344	230579286	DNER	delta and Notch-like epidermal growth factor-related receptor precursor
NM_004704	chr3	51967441	51975957	RRP9	U3 small nucleolar RNA-interacting protein 2
NR_132749	chr3	55542338	56502391	ERC2	ELKS/RAB6-interacting/CAST family member 2
NR_030293	chr3	114035321	114035416	MIR568	microRNA 568
NM_007064	chr3	124303505	124445172	KALRN	kalirin isoform 3
NR_073375	chr3	127317199	127341278	MCM2	minichromosome maintenance complex component 2
NM_003707	chr3	127799799	127842671	RUVBL1	ruvB-like 1 isoform 1
NM_007208	chr3	131181044	131221860	MRPL3	39S ribosomal protein L3, mitochondrial
NM_001080412	chr3	141043054	141168632	ZBTB38	zinc finger and BTB domain-containing protein 38
NM_003875	chr3	155588324	155655520	GMPS	GMP synthase [glutamine-hydrolyzing]
NM_017816	chr4	4269428	4291896	LYAR	cell growth-regulating nucleolar protein
NR_026854	chr4	39481874	39483523	LOC401127	WD repeat domain 5 pseudogene

Supplementary Table 3. TIN-signature genes.

Table with the RefSeq accession, chromosomal position and brief description of the 184 genes included in the TIN-signature listed by chromosome and position.

RefSeq_Accession	Chromosome	Start	End	Symbol	Description
NM_001079525	chr4	57302268	57327534	PAICS	multifunctional protein ADE2 isoform 1
NM_032313	chr4	57829509	57843826	NOA1	nitric oxide-associated protein 1
NM_032993	chr4	110736665	110745893	GAR1	H/ACA ribonucleoprotein complex subunit 1
NM_002358	chr4	120980578	120988013	MAD2L1	mitotic spindle assembly checkpoint protein MAD2A
NM_001302694	chr4	154178510	154197427	TRIM2	tripartite motif-containing protein 2 isoform 5
NR_045605	chr4	154265800	154336247	MND1	meiotic nuclear divisions 1
NM_018352	chr4	164415672	164441691	TMA16	translation machinery-associated protein 16
NR_102746	chr5	10440846	10441963	ROPN1L-AS1	ROPN1L antisense RNA 1
NM_031966	chr5	68462836	68474070	CCNB1	G2/mitotic-specific cyclin-B1
NM_014819	chr5	108670409	108745675	PJA2	E3 ubiquitin-protein ligase Praja-2
NM_001017398	chr5	114513235	114516243	TRIM36	E3 ubiquitin-protein ligase TRIM36 isoform 3
NM_003059	chr5	131630144	131679899	SLC22A4	solute carrier family 22 member 4
NM_032289	chr5	139175405	139224048	PSD2	PH and SEC7 domain-containing protein 2
NM_030571	chr5	141488323	141534008	NDFIP1	NEDD4 family-interacting protein 1
NM_006638	chr6	4995279	5004297	RPP40	ribonuclease P protein subunit p40 isoform a
NM_001128164	chr6	16299342	16761721	ATXN1	ataxin-1
NR_015410	chr6	21666674	22194616	CASC15	cancer susceptibility candidate 15
NM_005319	chr6	26055967	26056699	HIST1H1C	histone H1.2
NM_021904	chr6	29570004	29600912	GABBR1	gamma-aminobutyric acid type B receptor subunit 1 isoform c precursor
NM_015245	chr6	34857037	35059190	ANKS1A	ankyrin repeat and SAM domain-containing protein 1A
NM_153487	chr6	37600283	37665766	MDGA1	MAM domain-containing glycosylphosphatidylinositol anchor protein 1 precursor
NM_015349	chr6	42788793	42836298	GLTSCR1L	GLTSCR1-like protein
NM_018368	chr6	70385640	70507049	LMBRD1	probable lysosomal cobalamin transporter
NM_015018	chr6	83777384	83878190	DOPEY1	protein dopey-1 isoform a
NM_153816	chr6	86215214	86303874	SNX14	sorting nexin-14 isoform a
NM_018013	chr6	107811316	107982513	SOBP	sine oculis-binding protein homolog
NM_002912	chr6	111620233	111804918	REV3L	DNA polymerase zeta catalytic subunit isoform a
NM_020755	chr6	122764492	122793026	SERINC1	serine incorporator 1 precursor
NM_001134832	chr6	135708921	135818903	AHI1	jouberin isoform b
NM_003980	chr6	136663418	136871957	MAP7	ensconsin isoform 3
NM_006734	chr6	143072603	143266338	HIVEP2	transcription factor HIVEP2
NM_152745	chr7	8473584	8792593	NXPH1	neurexophilin-1 precursor
NM_018374	chr7	12250847	12276890	TMEM106B	transmembrane protein 106B
NM_015052	chr7	43152197	43602938	HECW1	E3 ubiquitin-protein ligase HECW1 isoform a
NR_133927	chr7	54613856	54638773	VSTM2A	V-set and transmembrane domain containing 2A
NM_001256414	chr7	79765070	79848725	GNAI1	guanine nucleotide-binding protein G(i) subunit alpha-1 isoform 2
NM_138290	chr7	87257728	87461613	RUNDC3B	RUN domain-containing protein 3B isoform a
NM_022900	chr7	94139169	94186328	CASD1	CAS1 domain-containing protein 1 precursor
NM_014705	chr7	111366163	111846462	DOCK4	dedicator of cytokinesis protein 4
NM_182597	chr7	112121065	112130943	LSMEM1	leucine-rich single-pass membrane protein 1
NM_024429	chr7	151253200	151329344	PRKAG2	5'-AMP-activated protein kinase subunit gamma-2 isoform b
NM_181723	chr8	16884746	16980148	MICU3	calcium uptake protein 3, mitochondrial
NM_007029	chr8	80523048	80578410	STMN2	stathmin-2 isoform 2
NM_024094	chr8	120846180	120868170	DSCC1	sister chromatid cohesion protein DCC1
NR_104612	chr9	37753799	37778969	TRMT10B	tRNA methyltransferase 10B
NR_131751	chr9	79226291	79521003	PRUNE2	prune homolog 2
NM_001827	chr9	91926109	91931618	CKS2	cyclin-dependent kinases regulatory subunit 2
NM_014142	chr10	12207326	12238143	NUDT5	ADP-sugar pyrophosphatase isoform 1
NR_110297	chr10	14946609	14996431	DCLRE1C	DNA cross-link repair 1C
NM_003635	chr10	75561668	75571589	NDST2	bifunctional heparan sulfate N-deacetylase/N-sulfotransferase 2 isoform 1
NM_001284242	chr10	86184937	86278277	CCSER2	serine-rich coiled-coil domain-containing protein 2 isoform 4
NM_014912	chr10	93808396	94050875	CPEB3	cytoplasmic polyadenylation element-binding protein 3 isoform 1
NM_138421	chr11	18101889	18127638	SAAL1	protein SAAL1
NM_002804	chr11	47440319	47448024	PSMC3	26S protease regulatory subunit 6A
NM_033396	chr11	57067102	57092413	TNKS1BP1	182 kDa tankyrase-1-binding protein
NM_004111	chr11	61560108	61564714	FEN1	flap endonuclease 1
NM_001667	chr11	64781584	64789657	ARL2	ADP-ribosylation factor-like protein 2 isoform 1
NM_080668	chr11	64844926	64851615	CDCA5	sororin
NM_182710	chr11	65479472	65487077	KAT5	histone acetyltransferase KAT5 isoform 1
NM_173582	chr11	74041356	74109510	PGM2L1	glucose 1,6-bisphosphate synthase
NR_126004	chr11	112141471	112233257	LOC283140	uncharacterized LOC283140
NM_001143668	chr12	47470382	47473734	AMIGO2	amphoterin-induced protein 2 precursor
NM_006313	chr12	62654120	62803501	USP15	ubiquitin carboxyl-terminal hydrolase 15 isoform 2

Supplementary Table 3. TIN-signature genes.

RefSeq_Accession	Chromosome	Start	End	Symbol	Description
NR_037687	chr12	94656296	94701451	PLXNC1	plexin C1
NM_152726	chr13	22066827	22178355	MICU2	calcium uptake protein 2, mitochondrial
NM_001195430	chr13	36420036	36429998	DCLK1	serine/threonine-protein kinase DCLK1 isoform 4
NR_024458	chr13	45915479	45965618	TPT1-AS1	TPT1 antisense RNA 1
NM_012158	chr13	77579388	77601331	FBXL3	F-box/LRR-repeat protein 3
NM_001320854	chr13	111893491	111947542	ARHGEF7	rho guanine nucleotide exchange factor 7 isoform g
NR_120600	chr14	23388664	23392616	PRMT5-AS1	PRMT5 antisense RNA 1
NR_051979	chr14	24649424	24658124	IPO4	importin 4
NM_005192	chr14	54863672	54886934	CDKN3	cyclin-dependent kinase inhibitor 3 isoform 1
NM_003384	chr14	97263683	97347951	VRK1	serine/threonine-protein kinase VRK1
NM_021709	chr14	105219469	105225996	SIVA1	apoptosis regulatory protein Siva isoform 2
NM_145701	chr14	105475909	105487425	CDCA4	cell division cycle-associated protein 4
NM_014985	chr15	49030134	49103343	CEP152	centrosomal protein of 152 kDa isoform 2
NM_018080	chr15	62156504	62352664	VPS13C	vacuolar protein sorting-associated protein 13C isoform 1B
NM_148955	chr15	64388082	64436433	SNX1	sorting nexin-1 isoform b
NM_006901	chr15	72118360	72410440	MYO9A	unconventional myosin-IXa
NM_001286440	chr16	447741	450754	NME4	nucleoside diphosphate kinase, mitochondrial isoform d
NR_130754	chr16	11439294	11445620	RMI2	RecQ mediated genome instability 2
NR_135304	chr16	30204250	30204868	BOLA2	bolA family member 2
NR_134471	chr16	30435018	30441121	DCTPP1	dCTP pyrophosphatase 1
NM_007006	chr16	56463047	56485261	NUDT21	cleavage and polyadenylation specificity factor subunit 5
NR_074074	chr16	69775756	69788871	NOB1	NIN1/PSMD8 binding protein 1 homolog
NM_030928	chr16	88870185	88875666	CDT1	DNA replication factor Cdt1
NM_015113	chr17	3907738	4046253	ZZEF1	zinc finger ZZ-type and EF-hand domain-containing protein 1
NR_106924	chr17	4872996	4873066	MIR6864	microRNA 6864
NM_001162371	chr17	5402746	5404319	LOC728392	uncharacterized protein LOC728392
NM_020899	chr17	7362684	7387568	ZBTB4	zinc finger and BTB domain-containing protein 4
NM_003010	chr17	11924134	12047148	MAP2K4	dual specificity mitogen-activated protein kinase kinase 4 isoform 1
NM_001330143	chr17	15138535	15168690	PMP22	peripheral myelin protein 22 isoform 2
NM_020652	chr17	15602890	15624100	ZNF286A	zinc finger protein 286A isoform 2
NM_001288789	chr17	17746821	17875784	TOM1L2	TOM1-like protein 2 isoform 7
NM_148921	chr17	19140689	19240028	EPN2	epsin-2 isoform a
NM_001031806	chr17	19552063	19580904	ALDH3A2	fatty aldehyde dehydrogenase isoform 1
NM_014683	chr17	19674142	19771239	ULK2	serine/threonine-protein kinase ULK2
NM_052888	chr17	30348154	30380519	LRRC37B	leucine-rich repeat-containing protein 37B isoform 1 precursor
NM_001254	chr17	38444145	38459413	CDC6	cell division control protein 6 homolog
NM_006695	chr17	42385926	42395238	RUNDC3A	RUN domain-containing protein 3A isoform 2
NM_153228	chr17	54230835	54560007	ANKFN1	ankyrin repeat and fibronectin type-III domain-containing protein 1
NM_003835	chr17	63133455	63223821	RGS9	regulator of G-protein signaling 9 isoform 1
NM_015167	chr17	74714524	74722881	JMJD6	bifunctional arginine demethylase and lysyl-hydroxylase JMJD6 isoform 2
NM_012307	chr18	5392379	5544309	EPB41L3	band 4.1-like protein 3 isoform 1
NR_003688	chr19	10220424	10220516	SNORD105B	small nucleolar RNA, C/D box 105B
NM_006397	chr19	12917427	12924462	RNASEH2A	ribonuclease H2 subunit A
NM_012321	chr19	18417039	18434001	LSM4	U6 snRNA-associated Sm-like protein LSm4 isoform 1
NM_013348	chr19	48958963	48969367	KCNJ14	ATP-sensitive inward rectifier potassium channel 14
NM_017916	chr19	49949549	49955115	PIH1D1	PIH1 domain-containing protein 1
NM_001009984	chr20	3229947	3388309	C20orf194	uncharacterized protein C20orf194
NM_021156	chr20	7957999	8000476	TMX4	thioredoxin-related transmembrane protein 4 precursor
NM_152227	chr20	17922239	17949634	SNX5	sorting nexin-5 isoform a
NM_005225	chr20	32263291	32274210	E2F1	transcription factor E2F1
NM_001322086	chr20	32868070	32890812	AHCY	adenosylhomocysteinase isoform 3
NR_002165	chr20	33421377	33422265	HMGB3P1	high mobility group box 3 pseudogene 1
NM_001278641	chr22	20105475	20114880	RANBP1	ran-specific GTPase-activating protein isoform 4
NM_145862	chr22	29083730	29137822	CHEK2	serine/threonine-protein kinase Chk2 isoform b
NM_006746	chrX	17755568	17773108	SCML1	sex comb on midleg-like protein 1 isoform b
NM_006406	chrX	23685644	23704514	PRDX4	peroxiredoxin-4 precursor
NM_016937	chrX	24712063	25015102	POLA1	DNA polymerase alpha catalytic subunit isoform 2
NM_001166704	chrX	55171677	55187628	FAM104B	protein FAM104B isoform 7
NM_012310	chrX	69509878	69640774	KIF4A	chromosome-associated kinesin KIF4A
NM_012278	chrX	70521597	70525221	ITGB1BP2	integrin beta-1-binding protein 2 isoform 1
NM_002764	chrX	106871653	106894256	PRPS1	ribose-phosphate pyrophosphokinase 1 isoform 1
NM_001301228	chrX	150151857	150159248	HMGB3	high mobility group protein B3 isoform a
NM_003491	chrX	153195279	153200607	NAA10	N-alpha-acetyltransferase 10 isoform 1

Supplementary Table 3. TIN-signature genes.

	Stage	AgeDiagnosis	TIN-index	TIN-Sign.	MYCN	Risk-group
Stage	-	0.575	0.041 *	0.107	<0.001 ***	0.611
AgeDiagnosis	0.580	-	0.039 *	0.079	0.002 **	0.780
TIN-index	0.045 *	0.041 *	-	0.523	<0.001 ***	0.025 *
TIN-Sign.	0.110	0.081	0.527	-	<0.001 ***	0.069
MYCN	<0.001 ***	0.002 **	<0.001 ***	<0.001 ***	-	<0.001 ***
Risk-group	0.611	0.781	0.024 *	0.070	<0.001 ***	-

Supplementary Table 4. ROC curves test.

The p-values of the test performed on the ROC curves of Fig.2A. The table shows the p-values of the test performed for all pairwise comparisons' of the mentioned ROC curves. In the upper triangle are reported the p-values using the 'bootstrap' method in the 'roc.test' function, while in the lower triangle are reported the p-values using the default 'delong' method. Significant results are followed by stars: ***) when the p-value was lower than 0.001, **) with p-values between 0.001 and lower than 0.01 and *) with p-values between 0.01 and lower than 0.05. The TIN-index AUC resulted to be significantly bigger when compared to the AUCs of all the known prognostic factors (according to both methods used for the bootstrapping), while it was not different from the TIN-signature one.

Database	Database_ID	Pathway_CanonicalName	Count: genes in Pathway	Count: genes in TIN-signature	Corrected enrichment p-value (Westfall-Young minP)	Uncorrected enrichment p-value (Fisher exact)
KEGG	4110	Cell cycle	124	7	$<1.00x10^{-4}$	$6.91x10^{-8}$
KEGG	230	*Purine metabolism	163	7	$<1.00x10^{-4}$	$4.46x10^{-7}$
KEGG	3030	**DNA replication	36	4	$1.00x10^{-4}$	$3.29x10^{-6}$
KEGG	1100	Metabolic pathways	1131	12	$5.70x10^{-3}$	$9.32x10^{-5}$
KEGG	3450	***Non-homologous end-joining	13	2	$3.83x10^{-2}$	$5.98x10^{-4}$
NCI	500226	*Purine ribonucleoside monophosphate biosynthesis	7	3	$1.00x10^{-4}$	$7.54x10^{-7}$
NCI	200032	**E2F transcription factor network	72	5	$3.00x10^{-4}$	$1.97x10^{-6}$
NCI	200142	**FOXM1 transcription factor network	39	4	$4.00x10^{-4}$	$4.56x10^{-6}$
NCI	500386	**Assembly of the pre-replicative complex	14	3	$6.00x10^{-4}$	$7.73x10^{-6}$
NCI	500981	**Activation of the pre-replicative complex	22	3	$5.40x10^{-3}$	$3.21x10^{-5}$
NCI	500987	**Association of licensing factors with the pre-replicative complex	5	2	$1.37x10^{-2}$	$7.79x10^{-5}$
NCI	200072	***ATM pathway	34	3	$2.12x10^{-2}$	$1.22x10^{-4}$
NCI	500996	***Inhibition of replication initiation of damaged DNA by Rb/E2F1	7	2	$2.78x10^{-2}$	$1.63x10^{-4}$
NCI	501024	**G1/S Transition	8	2	$3.46x10^{-2}$	$2.17x10^{-4}$
NCI	500869	**CDT1 association with the CDC6:ORC:origin complex	44	3	$3.71x10^{-2}$	$2.64x10^{-4}$
NCI	500892	**Orc1 removal from chromatin	48	3	$4.45x10^{-2}$	$3.42x10^{-4}$
NCI	500964	**Removal of licensing factors from origins	50	3	$4.84x10^{-2}$	$3.86x10^{-4}$
Reactome	REACT_152	Cell Cycle, Mitotic	282	12	$<1.00x10^{-4}$	$3.47x10^{-11}$
Reactome	REACT_383	**DNA replication	181	8	$<1.00x10^{-4}$	$5.26x10^{-8}$
Reactome	REACT_1538	Cell Cycle Checkpoints	101	6	$<1.00x10^{-4}$	$4.54x10^{-7}$
Reactome	REACT_22172	Chromosome Maintenance	71	4	$1.10x10^{-3}$	$5.02x10^{-5}$
Reactome	REACT_1698	*Metabolism of nucleotides	76	4	$1.30x10^{-3}$	$6.56x10^{-5}$

Supplementary Table 5. Summary of the pathway enrichment analysis on the TIN-signature genes.

Table summarizing the pathway enrichment analysis using three different pathway databases. The pathways reported were selected to have a Westfall-Young p-value <0.05. *=pathways correlated to replication metabolism; **=pathways correlated to replication initiation and progression; ***=pathways correlated to replication stress management.

KEGG_ID	Pathway_CanonicalName	Count: genes in Pathway	Count: genes in TIN-signature	Corrected enrichment p-value (Westfall-Young minP)	Uncorrected enrichment p-value (Fisher exact)
4110	Cell cycle	124	7	$<1.00\times10^{-4}$	6.91×10^{-8}
230	Purine metabolism	163	7	$<1.00\times10^{-4}$	4.46×10^{-7}
3030	DNA replication	36	4	1.00×10^{-4}	3.29×10^{-6}
1100	Metabolic pathways	1131	12	5.70×10^{-3}	9.32×10^{-5}
3450	Non-homologous end-joining	13	2	3.83×10^{-2}	5.98×10^{-4}
3008	Ribosome biogenesis in eukaryotes	81	3	8.93×10^{-2}	1.58×10^{-3}
4914	Progesterone-mediated oocyte maturation	86	3	1.02×10^{-1}	1.87×10^{-3}
5219	Bladder cancer	42	2	2.96×10^{-1}	6.26×10^{-3}
4145	Phagosome	153	3	4.22×10^{-1}	9.34×10^{-3}
5223	Non-small cell lung cancer	54	2	4.54×10^{-1}	1.02×10^{-2}
330	Arginine and proline metabolism	55	2	4.67×10^{-1}	1.05×10^{-2}
471	D-Glutamine and D-glutamate metabolism	4	1	4.93×10^{-1}	1.12×10^{-2}
5214	Glioma	65	2	5.52×10^{-1}	1.45×10^{-2}
4115	p53 signaling pathway	68	2	5.74×10^{-1}	1.58×10^{-2}
5212	Pancreatic cancer	70	2	6.05×10^{-1}	1.67×10^{-2}
3018	RNA degradation	71	2	6.36×10^{-1}	1.71×10^{-2}
5218	Melanoma	71	2	6.36×10^{-1}	1.71×10^{-2}
5220	Chronic myeloid leukemia	73	2	6.44×10^{-1}	1.81×10^{-2}
4144	Endocytosis	201	3	6.62×10^{-1}	1.93×10^{-2}
5222	Small cell lung cancer	85	2	7.37×10^{-1}	2.40×10^{-2}
5215	Prostate cancer	89	2	7.62×10^{-1}	2.61×10^{-2}
4540	Gap junction	90	2	7.66×10^{-1}	2.67×10^{-2}
240	Pyrimidine metabolism	100	2	8.02×10^{-1}	3.24×10^{-2}
4912	GnRH signaling pathway	101	2	8.06×10^{-1}	3.30×10^{-2}
5142	Chagas disease (American trypanosomiasis)	104	2	8.16×10^{-1}	3.48×10^{-2}
4114	Oocyte meiosis	112	2	8.42×10^{-1}	3.98×10^{-2}
670	One carbon pool by folate	18	1	9.07×10^{-1}	4.93×10^{-2}

Supplementary Table 6. KEGG pathways enrichment analysis on the TIN-signature. Table with pathway enrichment analysis results using the KEGG pathway database. The pathways reported were selected to have a Fisher exact p-value <0.05.

NCI_ID	Pathway_CanonicalName	Count: genes in Pathway	Count: genes in TIN-signature	Corrected enrichment p-value (Westfall-Young minP)	Uncorrected enrichment p-value (Fisher exact)
500226	Purine ribonucleoside monophosphate biosynthesis	7	3	1.00×10^{-4}	7.54×10^{-7}
200032	E2F transcription factor network	72	5	3.00×10^{-4}	1.97×10^{-6}
200142	FOXM1 transcription factor network	39	4	4.00×10^{-4}	4.56×10^{-6}
500386	Assembly of the pre-replicative complex	14	3	6.00×10^{-4}	7.73×10^{-6}
500981	Activation of the pre-replicative complex	22	3	5.40×10^{-3}	3.21×10^{-5}
500987	Association of licensing factors with the pre-replicative complex	5	2	1.37×10^{-2}	7.79×10^{-5}
200072	ATM pathway	34	3	2.12×10^{-2}	1.22×10^{-4}
500996	Inhibition of replication initiation of damaged DNA by Rb/E2F1	7	2	2.78×10^{-2}	1.63×10^{-4}
501024	G1/S Transition	8	2	3.46×10^{-2}	2.17×10^{-4}
500869	CDT1 association with the CDC6:ORC:origin complex	44	3	3.71×10^{-2}	2.64×10^{-4}
500892	Orc1 removal from chromatin	48	3	4.45×10^{-2}	3.42×10^{-4}
500964	Removal of licensing factors from origins	50	3	4.84×10^{-2}	3.86×10^{-4}
200017	p73 transcription factor network	73	3	1.13×10^{-1}	1.17×10^{-3}
200056	Validated targets of C-MYC transcriptional activation	81	3	1.27×10^{-1}	1.58×10^{-3}
200111	C-MYC pathway	22	2	1.46×10^{-1}	1.74×10^{-3}
500684	5-Phosphoribose 1-diphosphate biosynthesis	1	1	3.62×10^{-1}	2.81×10^{-3}
200195	Aurora A signaling	31	2	3.98×10^{-1}	3.45×10^{-3}
200194	Regulation of RAC1 activity	38	2	4.45×10^{-1}	5.15×10^{-3}
200038	ATR signaling pathway	39	2	4.56×10^{-1}	5.42×10^{-3}
500840	Adenylate cyclase inhibitory pathway	2	1	6.62×10^{-1}	5.61×10^{-3}
500935	G1/S-Specific Transcription	2	1	6.62×10^{-1}	5.61×10^{-3}
500327	Inhibition of the proteolytic activity of APC/C required for the onset of	2	1	6.62×10^{-1}	5.61×10^{-3}
500325	Amplification of signal from unattached kinetochores via a MAD2 inhibi	2	1	6.62×10^{-1}	5.61×10^{-3}
500373	E2F mediated regulation of DNA replication	2	1	6.62×10^{-1}	5.61×10^{-3}
200190	PAR1-mediated thrombin signaling events	42	2	6.73×10^{-1}	6.26×10^{-3}
500976	Mitotic Prometaphase	43	2	6.78×10^{-1}	6.56×10^{-3}
500925	CDK-mediated phosphorylation and removal of Cdc6	43	2	6.78×10^{-1}	6.56×10^{-3}
200120	Direct p53 effectors	135	3	6.78×10^{-1}	6.64×10^{-3}
200064	Integrin-linked kinase signaling	45	2	6.88×10^{-1}	7.16×10^{-3}
500932	Switching of origins to a post-replicative state	47	2	6.98×10^{-1}	7.79×10^{-3}
501019	Activation of PUMA and translocation to mitochondria	3	1	8.13×10^{-1}	8.40×10^{-3}
500724	Early Phase of HIV Life Cycle	3	1	8.13×10^{-1}	8.40×10^{-3}
500397	APC/C:Cdc20 mediated degradation of Cyclin B	3	1	8.13×10^{-1}	8.40×10^{-3}
500939	G2/M DNA replication checkpoint	3	1	8.13×10^{-1}	8.40×10^{-3}
500988	Activation of NOXA and translocation to mitochondria	3	1	8.13×10^{-1}	8.40×10^{-3}
500382	Recruitment of NuMA to mitotic centrosomes	3	1	8.13×10^{-1}	8.40×10^{-3}
500260	Activation of BH3-only proteins	3	1	8.13×10^{-1}	8.40×10^{-3}
200121	Regulation of Androgen receptor activity	52	2	8.20×10^{-1}	9.47×10^{-3}
500392	Phosphorylation of Emi1	4	1	8.86×10^{-1}	1.12×10^{-2}
500396	APC/C:Cdc20 mediated degradation of mitotic proteins	4	1	8.86×10^{-1}	1.12×10^{-2}
200225	Signaling events mediated by focal adhesion kinase	58	2	8.88×10^{-1}	1.17×10^{-2}
200207	p53 pathway	59	2	8.90×10^{-1}	1.21×10^{-2}
500384	Loss of Nlp from mitotic centrosomes	61	2	8.92×10^{-1}	1.29×10^{-2}
500383	Loss of proteins required for interphase microtubule organization	61	2	8.92×10^{-1}	1.29×10^{-2}
500380	Centrosome maturation	61	2	8.92×10^{-1}	1.29×10^{-2}
500672	Removal of the Flap Intermediate from the C-strand	5	1	9.27×10^{-1}	1.40×10^{-2}
500719	AMPK inhibits chREBP transcriptional activation activity	5	1	9.27×10^{-1}	1.40×10^{-2}
500992	Removal of the Flap Intermediate	5	1	9.27×10^{-1}	1.40×10^{-2}
200218	PLK3 signaling events	5	1	9.27×10^{-1}	1.40×10^{-2}
500318	Chk1/Chk2(Cds1) mediated inactivation of Cyclin B:Cdk1 complex	5	1	9.27×10^{-1}	1.40×10^{-2}
500360	Organic cation transport	5	1	9.27×10^{-1}	1.40×10^{-2}
500381	Recruitment of mitotic centrosome proteins and complexes	64	2	9.28×10^{-1}	1.41×10^{-2}
200224	Regulation of retinoblastoma protein	64	2	9.28×10^{-1}	1.41×10^{-2}
200069	CDC42 signaling events	70	2	9.37×10^{-1}	1.67×10^{-2}
500669	Telomere C-strand synthesis initiation	6	1	9.53×10^{-1}	1.67×10^{-2}
500667	Telomere Extension By Telomerase	6	1	9.53×10^{-1}	1.67×10^{-2}
500388	CDC6 association with the ORC:origin complex	6	1	9.53×10^{-1}	1.67×10^{-2}
500936	DNA replication initiation	6	1	9.53×10^{-1}	1.67×10^{-2}
500385	DNA Replication Pre-Initiation	6	1	9.53×10^{-1}	1.67×10^{-2}
500602	Import of palmitoyl-CoA into the mitochondrial matrix	6	1	9.53×10^{-1}	1.67×10^{-2}
500601	Activated AMPK stimulates fatty-acid oxidation in muscle	6	1	9.53×10^{-1}	1.67×10^{-2}
500431	Class C/3 (Metabotropic glutamate/pheromone receptors)	7	1	9.69×10^{-1}	1.95×10^{-2}
500374	E2F-enabled inhibition of pre-replication complex formation	7	1	9.69×10^{-1}	1.95×10^{-2}
200076	S1P5 pathway	8	1	9.78×10^{-1}	2.22×10^{-2}
500668	Telomere C-strand (Lagging Strand) Synthesis	9	1	9.80×10^{-1}	2.50×10^{-2}
500945	Removal of DNA patch containing abasic residue	9	1	9.80×10^{-1}	2.50×10^{-2}
500991	Cyclin A/B1 associated events during G2/M transition	10	1	9.82×10^{-1}	2.77×10^{-2}
500377	Unwinding of DNA	10	1	9.82×10^{-1}	2.77×10^{-2}
500078	Other semaphorin interactions	12	1	9.86×10^{-1}	3.32×10^{-2}
500857	mRNA Decay by 5' to 3' Exoribonuclease	13	1	9.89×10^{-1}	3.59×10^{-2}
500233	Synthesis and interconversion of nucleotide di- and triphosphates	13	1	9.89×10^{-1}	3.59×10^{-2}
200152	JNK signaling in the CD4+ TCR pathway	13	1	9.89×10^{-1}	3.59×10^{-2}
200134	ErbB1 downstream signaling	106	2	9.89×10^{-1}	3.60×10^{-2}
500109	Processing of Intronless Pre-mRNAs	14	1	9.93×10^{-1}	3.86×10^{-2}
500670	Polymerase switching on the C-strand of the telomere	14	1	9.93×10^{-1}	3.86×10^{-2}
200054	S1P4 pathway	14	1	9.93×10^{-1}	3.86×10^{-2}
200095	Arf6 downstream pathway	15	1	9.95×10^{-1}	4.13×10^{-2}
500855	Deadenylation of mRNA	16	1	9.96×10^{-1}	4.40×10^{-2}
200035	DNA-PK pathway in nonhomologous end joining	16	1	9.96×10^{-1}	4.40×10^{-2}
500395	Phosphorylation of the APC/C	17	1	9.97×10^{-1}	4.67×10^{-2}
200150	PDGFR-beta signaling pathway	126	2	9.97×10^{-1}	4.92×10^{-2}
200144	Hypoxic and oxygen homeostasis regulation of HIF-1-alpha	18	1	9.98×10^{-1}	4.93×10^{-2}
500328	Inactivation of APC/C via direct inhibition of the APC/C complex	18	1	9.98×10^{-1}	4.93×10^{-2}

Supplementary Table 7. NCI pathways enrichment analysis on the TIN-signature. Table with pathway enrichment analysis results using the NCI pathway database. The pathways reported were selected to have a Fisher exact p-value <0.05.

Reactome_ID	Pathway_CanonicalName	Count: genes in Pathway	Count: genes in TIN-signature	Corrected enrichment p-value (Westfall-Young minP)	Uncorrected enrichment p-value (Fisher exact)
REACT_152	Cell Cycle, Mitotic	282	12	$<1.00x10^{-4}$	$3.47x10^{-11}$
REACT_383	DNA Replication	181	8	$<1.00x10^{-4}$	$5.26x10^{-8}$
REACT_1538	Cell Cycle Checkpoints	101	6	$<1.00x10^{-4}$	$4.54x10^{-7}$
REACT_22172	Chromosome Maintenance	71	4	$1.10x10^{-3}$	$5.02x10^{-5}$
REACT_1698	Metabolism of nucleotides	76	4	$1.30x10^{-3}$	$6.56x10^{-5}$
REACT_21257	Metabolism of RNA	124	3	$8.07x10^{-2}$	$5.25x10^{-3}$
REACT_13685	Synaptic Transmission	183	3	$2.14x10^{-1}$	$1.51x10^{-2}$
REACT_18266	Axon guidance	232	3	$3.56x10^{-1}$	$2.80x10^{-2}$
REACT_216	DNA Repair	99	2	$4.11x10^{-1}$	$3.18x10^{-2}$
REACT_604	Hemostasis	424	4	$4.19x10^{-1}$	$3.21x10^{-2}$
REACT_1505	Integration of energy metabolism	106	2	$4.65x10^{-1}$	$3.60x10^{-2}$
REACT_11184	Botulinum neurotoxicity	17	1	$6.08x10^{-1}$	$4.67x10^{-2}$

Supplementary Table 8. Reactome pathways enrichment analysis on the TIN-signature.

Table with pathway enrichment analysis results using the Reactome pathway database. The pathways reported were selected to have a Fisher exact p-value <0.05.

Database	Database_ID	Pathway_CanonicalName	Higher expression in clusters with better prognosis	Higher expression in clusters with worse prognosis	Count: genes in Pathway	clusters with better prognosis: Count: genes in clusters	clusters with better prognosis: Uncorrected enrichment p-value (Fisher exact)	clusters with worse prognosis: Count: genes in clusters	clusters with worse prognosis: Uncorrected enrichment p-value (Fisher exact)
KEGG	4110	Cell cycle	no	yes	124	1	$1.47x10^{-1}$	5	$9.32x10^{-7}$
KEGG	5219	Bladder cancer **	no	yes	42	0	1	2	$1.60x10^{-3}$
KEGG	5223	Non-small cell lung cancer **	no	yes	54	0	1	2	$2.63x10^{-3}$
KEGG	5214	Glioma **	no	yes	65	0	1	2	$3.79x10^{-3}$
KEGG	5212	Pancreatic cancer **	no	yes	70	0	1	2	$4.38x10^{-3}$
KEGG	5218	Melanoma **	no	yes	71	0	1	2	$4.50x10^{-3}$
KEGG	5220	Chronic myeloid leukemia **	no	yes	73	0	1	2	$4.75x10^{-3}$
KEGG	3008	Ribosome biogenesis in eukaryotes	no	yes	81	0	1	2	$5.82x10^{-3}$
KEGG	5222	Small cell lung cancer **	no	yes	85	0	1	2	$6.39x10^{-3}$
KEGG	5215	Prostate cancer **	no	yes	89	0	1	2	$6.98x10^{-3}$
KEGG	240	Pyrimidine metabolism	no	yes	100	0	1	2	$8.74x10^{-3}$
KEGG	5142	Chagas disease (American trypanosomiasis)	no	yes	104	0	1	2	$9.42x10^{-3}$
KEGG	4360	Axon guidance	no	yes	129	0	1	2	$1.42x10^{-2}$
KEGG	4530	Tight junction	no	yes	132	0	1	2	$1.48x10^{-2}$
KEGG	3450	Non-homologous end-joining *	yes	no	13	2	$1.25x10^{-4}$	0	1
NCI	200032	E2F transcription factor network	no	yes	72	0	1	5	$6.13x10^{-8}$
NCI	500996	Inhibition of replication initiation of damaged DNA by Rb/E2F1	no	yes	7	0	1	2	$4.03x10^{-5}$
NCI	501024	G1/S Transition	no	yes	8	0	1	2	$5.37x10^{-5}$
NCI	200038	ATR signaling pathway	no	yes	39	0	1	2	$1.38x10^{-3}$
NCI	500976	Mitotic Prometaphase	no	yes	43	0	1	2	$1.68x10^{-3}$
NCI	200224	Regulation of retinoblastoma protein	no	yes	64	0	1	2	$3.68x10^{-3}$
NCI	200150	PDGFR-beta signaling pathway	no	yes	126	0	1	2	$1.36x10^{-2}$
NCI	200072	ATM pathway *	yes	no	34	2	$8.84x10^{-4}$	0	1
NCI	200017	p73 transcription factor network	yes	no	73	3	$1.18x10^{-4}$	0	1
NCI	500226	Purine ribonucleoside monophosphate biosynthesis	yes	no	7	2	$3.38x10^{-5}$	0	1
Reactome	REACT_22172	Chromosome Maintenance	no	yes	71	1	$8.70x10^{-2}$	3	$1.40x10^{-4}$
Reactome	REACT_604	Hemostasis	no	yes	424	1	$4.20x10^{-1}$	3	$2.18x10^{-2}$

Supplementary Table 9. Summary of the pathway enrichment analysis on the TIN-signature genes, stratified by patients' clusters.

Table summarizing the pathway enrichment analysis using three different pathway databases on two different groups of patients' clusters as identified at the top of Fig.2: a 'better prognosis' group with patients from the 'blue' and 'green' clusters, and a 'worse prognosis' group with patients from the 'red' and 'black' cluster. The pathways reported were selected to have a significant Fisher exact p-value of <0.05 on either of the two groups, with at least 2 genes mapping in the specified pathway. *=pathways related to DNA damage response; **=pathways related to cancer.

Panel a	Panel b	Panel c	Panel d	Panel e	Panel f	Panel g	Panel h
(chr1:5922731-9789172)	(chr2:11273178-20850864)	(chr2:27719705-33171202)	(chr2:30569511-38742882)	(chr20:49505454-56884495)	(chr17:39782578-40976310)	(chr10:35894337-45681489)	(chr11:9346368-10210415)
MIR4689	C2orf50	GCKR	LOC285043	ADNP	KRT42P	GJD4	SNORA25
NPHP4	PQLC3	C2orf16	LCLAT1	ADNP-AS1	EIF1	FZD8	SNORA32
KCNAB2	ROCK2	ZNF512	CAPN13	DPM1	GAST	MIR4683	SNORD6
CHD5	LINC00570	CCDC121	GALNT14	MOCS3	HAP1	PCAT5	SNORA1
RPL22	E2F6	GPN1	CAPN14	KCNG1	JUP	ANKRD30A	SNORA8
LOC102724450	GREB1	SUPT7L	EHD3	NFATC2	P3H4	LINC00993	SNORD5
RNF207	MIR4429	SLC4A1AP	XDH	MIR3194	FKBP10	MTRNR2L7	SNORA18
ICMT	GREB1	MRPL33	SRD5A2	ATP9A	NT5C3B	ZNF248	MIR1304
LINC00337	NTSR2	RBKS	MEMO1	SALL4	KLHL10	ZNF33BP1	SNORA40
HES3	LPIN1	BRE-AS1	DPY30	LINC01429	KLHL11	ZNF248	TAF1D
GPR153	MIR548S	BRE	SPAST	ZFP64	ACLY	LOC100129055	C11orf54
ACOT7	MIR4262	MIR4263	SLC30A6	LINC01524	TTC25	HSD17B7P2	MED17
HES2	LOC100506457	LOC100505736	NLRC4	TSHZ2	CNP	SEPT7P9	VSTM5
ESPN	MIR3681	LOC100505716	YIPF4	LOC101927770	DNAJC7	LINC00999	HEPHL1
MIR4252	TRIB2	FLJ31356	BIRC6	ZNF217	NKIRAS2	ACTR3BP5	PANX1
TNFRSF25	MIR3125	FOSL2	MIR558	SUMO1P1	ZNF385C	LOC441666	IZUMO1R
PLEKHG5	LOC100506474	PLB1	BIRC6-AS2	BCAS1	DHX58	CCNYL2	GPR83
NOL9	LINC00276	PPP1CB	TTC27	MIR4756	KAT2A	LINC00839	MRE11A
TAS1R1	FAM84A	SPDYA	MIR4765	CYP24A1	HSPB9	ZNF37BP	MIR548L
ZBTB48	LOC653602	TRMT61B	LINC00486	PFDN4	RAB5C	ZNF33B	ANKRD49
KLHL21	NBAS	WDR43	LOC100271832	DOK5	KCNH4	LINC01518	C11orf97
PHF13	DDX1	SNORD92	LTBP1	LINC01441	HCRT	BMS1	FUT4
THAP3	LOC101926966	SNORD53	MIR4430	LINC01440	GHDC	LINC01264	PIWIL4
DNAJC11	MYCNUT	FAM179A	RASGRP3	CBLN4	STAT5B	MIR5100	AMOTL1
LOC100505887	MYCNOS	C2orf71	FAM98A	MC3R	STAT5A	RET	CWC15
CAMTA1	MYCN	CLIP4	LINC01317	FAM210B	STAT3	CSGALNACT2	KDM4D
VAMP3	GACAT3	ALK	MYADML	AURKA	PTRF	RASGEF1A	KDM4E
PER3	FAM49A	YPEL5	LINC01320	CSTF1	ATP6V0A1	FXYD4	SRSF8
UTS2	RAD51AP2	LBH	LOC100288911	CASS4	MIR548AT	HNRNPF	ENDOD1
TNFRSF9	VSNL1	LOC285043	CRIM1	RTFDC1	MIR5010	ZNF487	LOC101929295
PARK7	SMC6	LCLAT1	FEZ2	GCNT7	NAGLU	ZNF239	SESN3
ERRFI1	GEN1	CAPN13	VIT	FAM209A	HSD17B1	ZNF485	LOC100129203
LOC102724539	MSGN1	GALNT14	STRN	FAM209B	COASY	ZNF32-AS3	FAM76B
SLC45A1	KCNS3	CAPN14	HEATR5B	TFAP2C	MLX	ZNF32	CEP57
RERE	RDH14	EHD3	GPATCH11	BMP7	PSMC3IP	ZNF32-AS1	MTMR2
LOC102724552	NT5C1B-RDH14	XDH	EIF2AK2	BMP7-AS1	FAM134C	ZNF32-AS2	MIR1260B
ENO1	NT5C1B	SRD5A2	SULT6B1	MIR4325	TUBG1	HNRNPA3P1	CCDC82
MIR6728	MIR4757	MEMO1	CEBPZOS	SPO11	TUBG2	LINC00619	JRKL
ENO1-AS1	OSR1	DPY30	CEBPZ	RAE1	PLEKHH3	LINC00840	JRKL-AS1
CA6	LINC00954	SPAST	NDUFAF7	MTRNR2L3	CCR10	LINC00841	CNTN5
SLC2A7	TTC32	SLC30A6	PRKD3	RBM38	CNTNAP1	C10orf142	ARHGAP42
SLC2A5	WDR35	NLRC4	QPCT	CTCFL	EZH1	CXCL12	TMEM133
GPR157	LOC101928222	YIPF4	CDC42EP3	PCK1	MIR6780A	TMEM72-AS1	PGR
MIR34A	MATN3	BIRC6	LINC00211	ZBP1	RAMP2-AS1	TMEM72	LOC101054525
H6PD	LAPTM4A	MIR558	RMDN2	PMEPA1	RAMP2	RASSF4	TRPC6
SPSB1	SDC1	BIRC6-AS2	RMDN2-AS1	NKILA	VPS25	C10orf10	MIR3920
LOC100506022	PUM2	TTC27	CYP1B1	MIR4532	WNK4	C10orf25	ANGPTL5
SLC25A33	RHOB	MIR4765	CYP1B1-AS1	C20orf85	COA3	ZNF22	CEP126
TMEM201	HS1BP3-IT1	LINC00486	ATL2	ANKRD60	CNTD1	RSU1P2	C11orf70
PIK3CD	HS1BP3	LOC100271832	LOC101929596	PPP4R1L	BECN1	ANKRD30BP3	YAP1

Supplementary Table 10. Genes mapping within the intervals represented in the local correlation heatmaps of Supplementary Figure 5.

Each column lists the 50 genes mapping within the corresponding interval related to the local correlation heatmaps of Supplementary Figure 5. Header row reports the panel ID, while the second row, between brackets, the related genomic coordinates are reported.
